# Phenolic Compounds from *Populus alba* L. and *Salix subserrata* Willd. (Salicaceae) Counteract Oxidative Stress in *Caenorhabditis elegans*

**DOI:** 10.3390/molecules24101999

**Published:** 2019-05-24

**Authors:** Nora Tawfeek, Mansour Sobeh, Dalia I. Hamdan, Nawaal Farrag, Mariana Roxo, Assem M. El-Shazly, Michael Wink

**Affiliations:** 1 Institute of Pharmacy and Molecular Biotechnology, Heidelberg University, Im Neuenheimer Feld 364, 69120 Heidelberg, Germany; noratawfeek1@gmail.com (N.T.); marianaroxocorreia@gmail.com (M.R.); 2 Department of Pharmacognosy, Faculty of Pharmacy, Zagazig University, Zagazig 44519, Egypt; nawalfarrag@yahoo.com (N.F.); assemels2002@yahoo.co.uk (A.M.E.-S.); 3 AgroBioSciences Research Division, Mohammed VI Polytechnic University, Lot 660–Hay MoulayRachid, 43150 Ben-Guerir, Morocco; 4 Department of Pharmacognosy, Faculty of Pharmacy, Menoufia University, Shibin Elkom 32511, Egypt; dalia_hamdan@yahoo.com

**Keywords:** Populus alba, Salix subserrata, Caenorhabditis elegans, oxidative stress

## Abstract

Utilizing bioassay- and TLC-guided column chromatography, fifteen secondary metabolites from *Populus alba* and eight compounds from *Salix subserrata* were isolated, including a novel plant metabolite salicyl ether and characterized using ultralviolet light (UV) absorbance, mass spectrometry (MS), ^1^H-, ^13^C-NMR (nuclear magnetic resonance), heteronuclear single quantum coherence spectroscopy (HSQC) and heteronuclear multiple bond correlation (HMBC). The extracts, their sub-fractions and the isolated compounds exhibited promising antioxidant activities in vitro in DPPH and FRAP assays. Also, the extracts of *P. alba* leaf (PL), shoots (PS), and *S. subserrata* leaf (SL) demonstrated substantial antioxidant activities in vivo in the multicellular model organism *Caenorhabditis elegans*. For the first time, the isolated secondary metabolites, aromadendrin, tremuloidin, salicin, isorhamnetin-3-*O*-β-d-rutinoside, gallocatechin, triandrin, and chrysoeriol-7-*O*-glucuronide were investigated. They exhibited substantial antioxidant activities in vivo. Salicin, isorhamnetin-3-*O*-β-d-rutinoside and gallocatechin, in particular, protected the worms against a lethal dose of the pro-oxidant juglone (80 µM), decreased the endogenous reactive oxygen species (ROS) level to 45.34%, 47.31%, 68.09% and reduced juglone- induced hsp-16.2::GFP (green fluorescence protein) expression to 79.62%, 70.17%, 26.77%, respectively. However, only gallocatechin induced higher levels of sod-3 expression. These findings support the traditional use of *Populus alba* and *Salix subserrata* for treating inflammation especially when ROS are involved.

## 1. Introduction

Oxidative stress is considered an imbalance between reactive oxygen species (ROS) and antioxidant defense mechanisms. Oxidative stress is either due to high production of ROS or retarded physiological antioxidant potential. Deleterious ROS including hydroxyl radical, hydrogen peroxide and superoxide anion are either products of endogenous metabolism or of exogenous factors such as hyperoxia, ionizing radiation, heavy metal ions, or smoking. These free radicals react with biological macromolecules causing oxidative damage and DNA mutations, which contribute to cancer, diabetes, atherosclerosis, hypertension, cardiovascular, inflammatory diseases, and others [1,2].

Cellular antioxidant defense mechanisms rely on some enzymes such as glutathione peroxidase, catalase, superoxide dismutase, and non-enzymatic substances like glutathione, vitamin A, C, and E. These physiological defenses are suppressed during pathological conditions. Great interest, therefore, has been directed towards investigating natural antioxidants in form of extracts or pure compounds from plants [3,4].

The Salicaceae family traditionally comprise *Salix* (willow) and *Populus* (poplar), which are common in northern temperate regions [5]. Numerous secondary metabolites were isolated from *P. alba* include flavonoids, salicin derivatives, phenolic acids, anthocyanins and polysaccharides [5,6]. As for *S. subserrata*, it was reported to produce flavonoids, salicin, phenolic alcohols, phenolic aldehyde and sterols [7,8].

*P. alba* exhibit various biological activities including in vitro antioxidant activity, cytotoxic and weak antibacterial activities [9,10]. From *S. subserrata*, cultivated in Egypt, in vitro antioxidant, anti-inflammatory, anticancer, hepatoprotective and antimicrobial activities were reported [11,12,13]. Willow bark is traditionally used to treat chronic pain, inflammation and fever.

The phytochemical composition of both *P. alba* and *S. subserrata* has not been thoroughly studied. In this work, we comprehensively isolated and characterized the secondary metabolites from *P. alba* and *S. subserrata*. The in vitro and in vivo antioxidant activities of the extracts and the isolated pure compounds were also evaluated. The widely used nematode *Caenorhabditis elegans* was used as an in vivo model [3,4].

## 2. Results and Discussion

### 2.1. Isolation and Structure Elucidation of the Isolated Compounds

By combining the bioassay-guided fractionation of the extracts, TLC-guided column chromatography and a series of different chromatographic techniques, compounds (**1**–**16**) were isolated from *P. alba* and (**17**–**24**) from *S. subserrata*. The corresponding structures were elucidated based on physiochemical and spectroscopic techniques as ultraviolet light (UV) absorbance, mass spectrometry (MS), ^1^H-and ^13^C-NMR (nuclear magnetic resonance), heteronuclear single quantum coherence spectroscopy (HSQC) and heteronuclear multiple bond correlation (HMBC).

**Figure 1:**
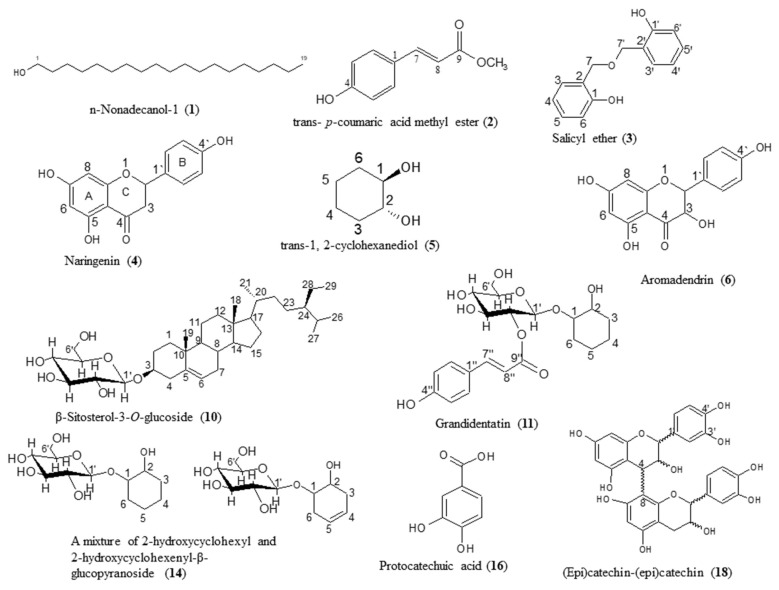

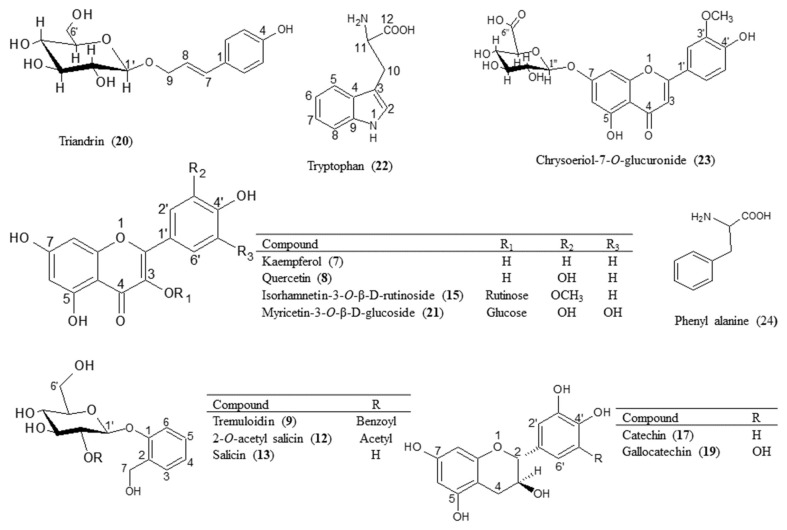
Structures of the isolated natural products from *P. alba* and *S. subserrata*.

The isolated compounds from *P. alba* were n-nonadecanol-1 (**1**) [14], trans *p*-coumaric acid methyl ester (**2**) [15], salicyl ether (**3**), naringenin (**4**) [16], trans-1,2 cyclohexanediol (**5**) [17,18], aromadendrin (**6**), kaempferol (**7**) [19], quercetin (**8**) [16], tremuloidin (**9**) [20,21], β-sitosterol 3-*O*-glucoside (**10**) [22], grandidentatin (**11**) [23], 2-*O*-acetyl salicin (**12**), salicin (**13**) [20,21], a mixture of 2-hydroxycyclohexyl-β-glucopyranoside and 2-hydroxycyclohexenyl-β-glucopyranoside (**14**) [24], isorhamnetin 3-*O*-β-d-rutinoside (**15**) [25], and protocatechuic acid (**16**) [26]. The purified compounds from *S. subserrata* are catechin (**17**) [7], (epi)catechin-(epi)catechin (**18**) [27], gallocatechin (**19**) [27], triandrin (**20**) [28], myricetin-3-*O*-β-d-glucoside (**21**) [29], tryptophan (**22**) [30], chrysoeriol-7-*O*-glucuronide (**23**) [31] and phenyl alanine (**24**) [32], Figure 1. UV, MS and NMR data of the isolated compounds from *Populus alba* L. and *Salix subserrata* Willd are included in Supplementary Materials.

**Figure 2:**
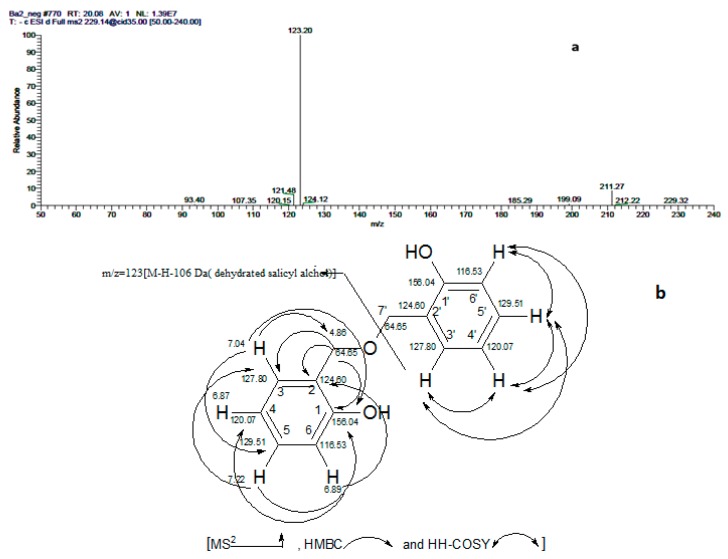
(**a**) MS^2^ (mass spectrometry) of compound (**3**), salicyl ether at [M − H]^−^ at *m/z* 229. (**b**) Proposed fragmentation pattern and selected nuclear magnetic resonance (NMR) correlations.

Compound **3**: Shiny colorless needles (30 mg), with R_f_ 0.4, solvent system (2), UV: **λ_max_** (MeOH) nm: 278. –ve electrospray ionization–mass spectrometry (ESI–MS), *m/z* (relative intensity %): 229 (100) [M − H]^−^ and *m/z* 459 (17) [2M − H]^−^, MS^2^ [229]: 211(6) [M − H − H_2_O]^−^, 123 (100) [M − H − 106 (dehydrated salicylalcohol)]^−^ and 121(5). +ve ESI–MS, *m/z* (relative intensity %): 253 (8) [M + Na]^+^, MS^2^ [253]: 213 (100). ^1^H-NMR (500 MHz, CDCl3): δ (ppm) 7.04 (2H, *dd*, *J* = 1.5, 7.5 Hz, H-3,3′), 6.87 (2H, *dt*, *J* = 1, 7.5 Hz, H-4, 4′), 7.22 (2H, *dt*, *J* = 1.5, 8 Hz, H-5,5′), 6.89 (2H, *dd*, *J* = 1,8 Hz, H-6, 6′) and 4.86 (4H, s, H-7, 7′). ^13^C-APT-NMR (125 MHz, CDCl3): δ(ppm) 156.04 (C-1,1′), 124.60 (C-2,2′), 127.80 (C-3,3′), 120.07 (C-4,4′), 129.51 (C-5,5′), 116.53 (C-6,6′) and 64.65 (C-7,7′). The HPLC-PDA provided the UV spectrum of compound (3) at maximum absorbance λ_max_ 278 nm, which closely matches that of hydroxy benzyl alcohol derivatives [20]. The –ve and +ve ESI–MS spectrum displayed product ion peaks of [M − H]^−^ at *m/z* 229 and 253 corresponding to [M − H]^−^ and [M + Na]^+^, respectively, suggesting a molecular formula of C_14_H_14_O_3_. The MS^2^ fragmentation of [M − H]^−^ ion at *m/z* 229 gave a base peak at *m/z* 123, corresponding to [salicyl alcohol − H]^-^, which was produced by the loss of dehydrated salicyl alcohol [M − H − 106]^−^, besides an ion at *m/z* 211 due to the loss of water molecule from the molecular ion peak [M − H − 18 (H_2_O)]^−^. In the down field region, ^1^H-NMR spectrum revealed four proton signals, each integrated for two protons, at δ_H_ 7.22 (*dt*, *J* = 1.5, 8 Hz, H-5, 5′), 7.04 (*dd*, *J* = 1.5, 7.5 Hz, H-3, 3′), 6.89 (*dd*, *J* = 1, 8 Hz, H-6, 6′) and 6.87 (*dt*, *J* = 1, 7.5 Hz, H-4,4′), indicating the presence of two (1,2-disubstituted aromatic ring). A sharp singlet appeared at δ_H_ 4.86, integrated for four oxygenated methylene protons. The ^13^C-APT-NMR spectrum revealed the presence of two quaternary carbon signals resonating at δ_C_ 156.04 ppm (C-1, 1′) and 124.60 ppm (C-2, 2′). In addition to, four signals for C-H aromatic carbons resonating at δ_C_ 129.51 (5,5′), 127.80 (3,3′), 120.07 (4, 4′) and 116.53 (6, 6′). The oxygenated methylenes C-7, 7′ exhibited more downfield peak at δ_C_ (64.65) rather than the free oxygenated methylene of salicyl alcohol (at δ 60.3) [33], which is evidence for its attachment. This pattern is typical for symmetrically bis (1,2-disubstituted benzene). Complete assignments of ^1^H- and ^13^C-APT-NMR signals were achieved by HH-COSY, HSQC and HMBC. The HSQC spectrum revealed that the singlet proton signal at δ_H_ 4.86 ppm (H-7, 7′) was directly attached to (C-7, 7′) carbons signal at δ_C_ 64.65 ppm. In addition, four ^13^C signals at δ_C_ 129.51, 127.80, 120.087 and 116.53 were directly attached to the ^1^H signals at δ_H_ 7.22 (*dt*, *J* = 1.5, 8 Hz, H-5, 5′), 7.04 (*dd*, *J* = 1.5, 7.5 Hz, H-3, 3′), 6.89 (*dd*, *J* = 1,8 Hz, H-6, 6′) and 6.87 (*dt*, *J* = 1,7.5 Hz, H-4, 4′) corresponding to (C-5, 5′), (C-3, 3′), (C-4, 4′) and (C-6, 6′), respectively, Figure 2. Thus, compound **3** was characterized as salicyl ether. As regards the current literature, it is the first isolation of this compound from a natural source.

### 2.2. Antioxidant Assays In Vitro

In this study, the in vitro antioxidant activities of *P. alba* leaf (PL), shoots (PS), *S. subserrata* leaf (SL) and stem bark (SB) extracts, their fractions and the purified compounds were assessed by two common DPPH and FRAP methods (Table 1 and Table 2). The total phenolic content was investigated using Folin-Ciocalteu method and it ranged from 25.37 ± 2.74 to 542.11 ± 4.88 mg gallic acid equivalent/g extract. Also, the total flavonoids content (TFC) was quantified using FeCl_3_ indicating that the extracts contain high amounts of flavonoids (Table 1). Noteworthy, *S*. *subserrata* stem bark extract showed the highest contents of phenolics and flavonoids and in DPPH assay as well (Table 1).

The antioxidant activity of the extracts and their fractions might be attributed to high level of flavonoids and phenolic acids, which are potent antioxidants, by virtue of their ability to scavenge free radicals as superoxide and hydroxyl radicals, and to chelate metal ions [34].

Compounds (**6**, **7**, **8**, **16**, **17**, **18**, **19**, **20**, and **21**) displayed strong activity and their activities in DPPH assay are ordered as **16** > **19** > **18** > **8** > **17** > **21** > **7** > **6** > **20** (Table 2). However, the rest of compounds demonstrated IC_50_ (the concentration which exhibited 50% scavenging for DPPH radicals) values above 100 µg/mL. Compound (**16**) was the most active compound due to presence of ortho-dihydroxyl groups (catechol type). These findings are consistent with the previously reported results [25,35,36,37,38,39,40,41].

The flavonoids (**6**, **7**, **8**, **17**, **18**, **19**, and **21**) displayed fairly strong antioxidant activity in comparison with epigallocatechin gallate (EGCG). Structure-activity relationship studies showed that the hydroxylation pattern of ring B is the most important factor for DPPH scavenging activity. Flavonoid with 3′, 4′dihydroxy substitution in ring B as compounds (**8**, **17**, **18**, and **19**) exhibited much higher activity than compounds (**6** and **7**) with only one free hydroxyl position 4′. The ortho-dihydroxy substitution in ring B ensures the stabilization of phenoxyl radical by formation of intramolecular hydrogen bonding and electron delocalization. Moreover, the metal chelation ability of the catechol moiety enhances the antioxidant potential. Another factor to aid the antioxidant activity is the presence of a 2, 3-double bond conjugated with the 4-oxo group, which enhances electron delocalization. This could explain why compound (**7**) (IC_50_ = 8.06 ± 0.07 µg/mL) is more active than compound (**6**) (IC_50_ = 18.64 ± 0.52 µg/mL). The presence of hydroxyl substitutions at C-3 and C-5 positions enhances the formation of stable quinone after flavonoid oxidation. Furthermore, the presence of a 3-hydroxyl group in ring C enhances the radical-scavenging activity which explains why compound (**6**) is more active (IC_50_ = 18.64 ± 0.52 µg/mL) than compound (**4**) (IC_50_ > 100 µg/mL) [25,35,41]. Salicin and its derivatives (compounds **9** and **12**) showed weak DPPH scavenging activity (IC_50_ > 100 µg/mL) due to the absence of free phenolic OH groups, similar results were obtained using ABTS^+^ radical [42] and ORAC assays [39].

In the FRAP assay, the antioxidant activity of the extracts, their fractions and the isolated compounds were assessed by their capacities to reduce Fe^+3^. The obtained results, as illustrated in Table 1 and Table 2 showed the same order of activity as that of DPPH assay, revealing reliable in vitro antioxidant activity of the studied extracts and their fractions. The previously reported in vitro antioxidant activity of *P*. *alba* [9], *S. subserrata* [11] and some isolated similar compounds [25,35,36,37,38,39,40,41] agree with the current work. Interestingly, a good linear correlation between total phenolic, total flavonoid profiles and antioxidant activities (DPPH and FRAP) was found when we compared the results from the four extracts (Table 3).

### 2.3. Antioxidant Assays In Vivo

The substantial in vitro antioxidant activities of the extracts and the major isolated compounds directed us to further investigate the in vivo antioxidant properties using the *C. elegans* model, which is widely used in this context [43,44,45]. Three different concentrations (50, 100 and 200 µg/mL) of the four extracts (PL, PS, SL and SB) were initially tested for their effect on survival rate of the wild-type N2 under juglone induced oxidative stress. The extract SB exhibited highest antioxidant activity in vitro, however it was toxic for the worms at the three mentioned doses (Table 1). This might be attributed to its high polyphenol content, among which are tannins, which can exhibit pro-oxidant activity at high concentrations as well as precipitate proteins [43,46]. Therefore, the further in vivo assays were included the three extracts (PL, PS and SL).

#### 2.3.1. Survival Assay under Oxidative Stress

**Figure 3:**
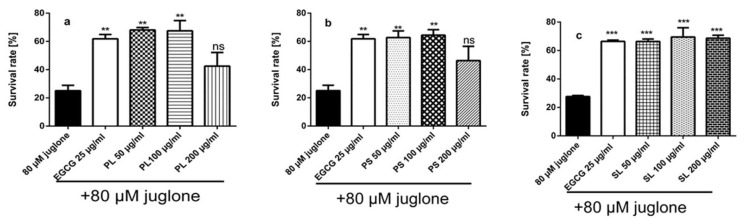
Effect of EGCG (epigallocatechin gallate) and extracts of PL (*P. alba* leaf), PS (*P. alba* shoots) and SL (*S. subserrata* leaf) on the survival rate of *C. elegans* N2 (wt) against a lethal dose of juglone (80 µM); (**a**) PL significantly enhanced the survival rate to 68.09% and 67.51% at doses of 50 and 100 µg/mL, respectively; (**b**) The survival rates were significantly improved after pre-treatment of the worms with 50 and 100 µg/mL of PS to 62.72% and 64.45%, respectively, while for the juglone treated control it was 25.08%; (**c**) SL significantly increased the survival rate to 66.36%, 69.52% and 68.65% at concentrations of 50, 100 and 200 µg/mL, respectively. The results are represented as mean ± standard error of the mean (SEM) from three independent experiments. ns (not significant) *p* > 0.05, ** *p* < 0.01, *** *p* < 0.0001, compared to untreated control (negative control) by one-way analysis of variance (ANOVA) followed by Bonferroni (post-hoc).

In the survival assay, N2 wild-type worms were pre-incubated with 50, 100 and 200 μg/mL extracts PL, PS, and SL for 48 h before being transferred to fresh medium and receiving a toxic dose of the pro-oxidant juglone (80 μM). The number of the surviving worms was scored 24 h later. The results showed that pre-treating the nematodes with 50 and 100 μg/mL extracts PL (Figure 3a) and PS (Figure 3b) protected the worms against oxidative stress when compared to the juglone treated control group. Interestingly, SL extract showed significant higher survival rate compared to the juglone treated control group with the three doses (50, 100 and 200 μg/mL) (Figure 3c).

#### 2.3.2. Intracellular Reactive Oxygen Species (ROS) Accumulation

**Figure 4:**
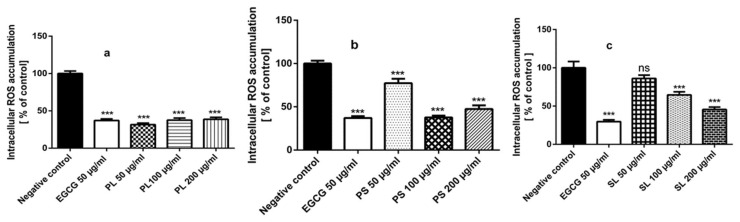
Influence of EGCG (epigallocatechin gallate) and the extracts of PL (*P. alba* leaf), PS (*P. alba* shoots) and SL (*S. subserrata* leaf) on intracellular ROS (reactive oxygen species) production in N2 worms, evidenced by the indicator H_2_DCF-DA. (**a**) PL significantly reduced ROS accumulation to 31.51%, 37.54% and 38.75% after pre-treatment with 50, 100 and 200 µg/mL, respectively. (**b**) PS treated N2 worms showed significant reduction in ROS level to 77.26%, 37.63% and 47.42% after pre-treatment with 50, 100 and 200 µg/mL, respectively. (**c**) ROS production was significantly reduced to 64.35% and 45.57% after pre-treatment with 100 and 200 µg/mL SL, respectively. The results are expressed as mean ± SEM from three independent experiments. Results are expressed as the percentage of fluorescence pixel intensity related to untreated control (100%). Statistical significance of difference between untreated control (negative control) and treated groups was analyzed by one-way analysis of variance followed by Bonferroni (post hoc) (ns (not significant) *p* > 0.05, *** *p* < 0.0001).

To assess the influence of the studied extracts on the intracellular ROS accumulation in vivo, N2 (wt) worms were incubated with 50, 100 and 200 μg/mL PL, PS and SL extracts for 48 h then they were exposed to the ROS indicator H_2_DCF-DA (6-carboxy-2′,7′-dichlorodihydrofluorescein diacetate) which is converted to deacetylated form (H_2_DCF) (6-carboxy-2′,7′- dichlorodihydrofluorescein) after passing through the cell membrane. The H_2_DCF is oxidized by intracellular reactive oxygen species to give oxidized form (DCF), which produces fluorescence. The intensity of the emitted fluorescence reflects the intracellular ROS contents [4]. The results demonstrated significant reduction (*p* < 0.0001) in ROS accumulation among the extracts PL (Figure 4a) and PS (Figure 4b) pre-treated worms when compared to the untreated control. Furthermore, pre-treating the worms with SL extract (100 and 200 µg/mL) demonstrated significant reduction in ROS levels in a dose-dependent manner (Figure 4c).

#### 2.3.3. Quantification of hsp-16.2::GFP (Green Fluorescence Protein) Expression and Subcellular DAF-16::GFP Localization

**Figure 5:**
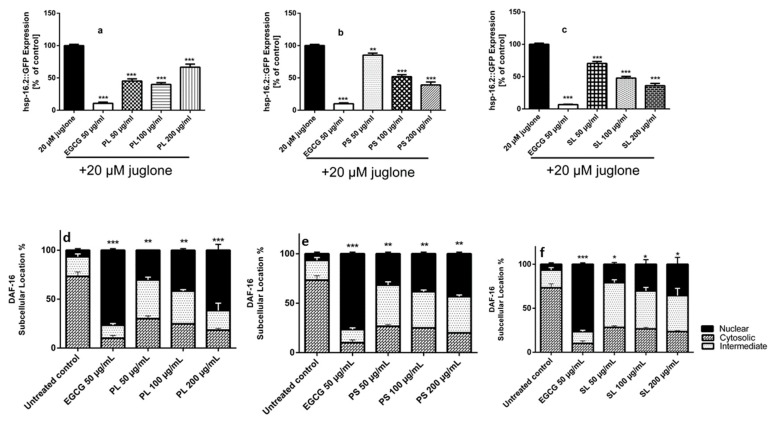
Effect of EGCG (epigallocatechin gallate) and extracts of PL (*P. alba* leaf), PS (*P. alba* shoots) and SL (*S. subserrata* leaf) on: hsp-16.2::GFP (green fluorescence protein) expression in the mutant strains TJ375 under juglone induced oxidative stress, juglone treated control was set 100% (**a**) The hsp-16.2::GFP expression levels was significantly reduced by 54.77%, 60.06% and 33.46% after pre-treatment of the worms with 50,100 and 200 µg/mL PL, respectively, followed by Juglone(20 µM). (**b**) PS significantly reduced hsp-16.2::GFP expression levels to 85.24%, 51.88% and 39.08% after pre-treatment of the worms with 50,100 and 200 µg/mL extract PS, respectively, followed by juglone(20 µM) (**c**) The hsp-16.2::GFP expression levels was significantly reduced by 29.55%, 52.43% and 64.03% after treatment of the worms with 50,100 and 200 µg/mL extract SL, respectively, followed by juglone (20 µM). Nuclear localization of DAF-16::GFP in the transgenic worms TJ356: (**d**) PL induced nuclear localization with 30.27, 41.73, and 61.77% by 50, 100, and 200 µg/mL respectively. (**e**) PS exerted subcellular nuclear translocation with 31.67, 38.33, 43.33% by 50, 100, and 200 µg/mL respectively. (**f**) SL demonstrated weak activities and activated DAF-16 with 21.00, 30.33, 35.67% by 50, 100, and 200 µg/mL respectively. EGCG was used a positive control. One-way ANOVA followed by Bonferroni (post –hoc) (** *p* < 0.01, *** *p* < 0.0001) was used for the analysis of statistical significance of differences between non-treated control and treated groups. The results are recorded as mean ± SEM from three independent experiments.

To get a clear insight about the involved mechanisms, we quantified heat shock proteins (HSPs) (hsp-16.2::GFP (green fluorescence protein)) expression after juglone treatment and the subcellular localization of DAF-16::GFP. HSPs are stress response proteins and are highly expressed in cells under thermal or oxidative stress [3,4]. These proteins are important for homeostasis of the living organisms and their actions are exerted by different mechanisms as inhibition of protein aggregation and enhancement of protein stabilization through assisting its folding and refolding [3,4]. The transgenic worm strains TJ375 that have the *hsp-16.2* gene connected with green fluorescence protein (GFP), were used to evaluate the effect of PL, PS and SL extracts on the expression of the stress marker hsp-16.2 under mild oxidative stress (20 µM juglone). Treatment of the transgenic strains TJ375 (hsp-16.2::GFP) with 20 µM juglone for 24 h induces free radical, over-expression of *hsp-16.2* gene and hence over-expression of GFP in the pharynx of transgenic worms TJ375 (hsp-16.2::GFP) [4]. The results showed that pre-treatment of the strains TJ375 with PL (Figure 5a), PS (Figure 5b) and SL (Figure 5c) extracts significantly reduced the expression of hsp-16.2 under oxidative stress. DAF-16 activates and regulates the expression of HSPs involved in longevity and oxidative stress responses. Figure 5 (d, e, and f) documents that the three extracts PL, PS, and SL were able to induce nuclear translocation of DAF-16::GFP in the transgenic worms TJ356. This indicates that DAF-16/FOXO may be involved in the antioxidant activities of the extracts.

#### 2.3.4. Quantification of Sod-3::GFP Expression

**Figure 6:**
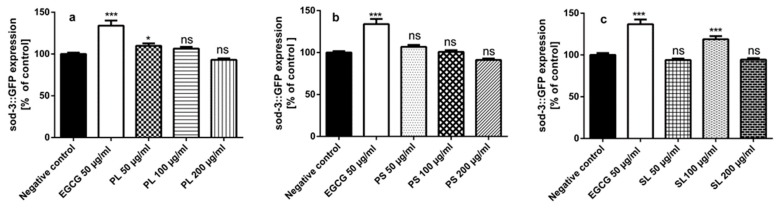
sod-3::GFP Expression in the mutant strains CF1553[(pAD76)sod-3::GFP+rol-6] after pre-treatment with the extracts, EGCG (epigallocatechin gallate) and extracts of PL (*P. alba* leaf), PS (*P. alba* shoots) and SL (*S. subserrata* leaf). (**a**) Pre-treated mutants CF1553 with 50 µg/mL PL exhibited higher levels of *sod-3* gene expression in comparison with control untreated worms. (**b**) PS showed no significant effect on sod-3 expression in mutants CF1553 compared to the untreated control. (**c**) Pre-treated worms CF1553 with 100 µg/mL SL showed higher levels of *sod-3* gene expression in comparing to control untreated worms. The results are presented as mean ± SEM (*n* = 3). ns (not significant) *p* > 0.05, * *p* < 0.05, *** *p* < 0.0001, analyzed by one-way ANOVA followed by Bonferroni (post –hoc).

For further investigation of the mechanisms by which PL, PS and SL extracts exhibit in vivo antioxidant activity, the expression of *sod-3* gene was assessed using the transgenic worm strains CF1553[(pAD76)sod-3::GFP+rol-6]. The *SOD-3* gene encodes for superoxide dismutase-3 (SOD-3) which is essential for oxidation-reduction balance. It converts superoxide anion by dismutation to hydrogen peroxide, which is then converted to oxygen and H_2_O by catalase and glutathione peroxidase [4]. Mutant strains CF1553 (sod-3::GFP) were incubated with the extracts PL, PS and SL for 72h and then the emitted fluorescence was measured. The results showed significant higher levels of sod-3 expression and higher fluorescence intensity among strains incubated with 50 µg/mL PL (Figure 6a) and 100 µg/mL SL (Figure 6c) extracts when compared to the untreated control group. There was no significance increase in sod-3 expression among PS (Figure 6b) extract treated *C. elegans* when compared to the untreated control.

Our findings imply the bioavailability of the extracts PL, PS and SL in *C. elegans* and these in vivo results clearly demonstrate that the components of the extracts are absorbed by the worms and have a vital role to improve the oxidative stress resistance in this animal model. Similar results have been described for other polyphenolic compounds and proanthocyanidins [47,48].

### 2.4. In Vivo Antioxidant Assays of Purified Compounds

The substantial antioxidant activities from the extracts directed us to further explore their individual constituents. In this attempt, we shed a light on the in vivo antioxidant activities of compounds (**6**, **9**, **13**, **15**, **19**, **20**, and **23**) by employing the multicellular model organism *C. elegans*. The rest of the compounds have been examined before. See quercetin, naringenin and kaempferol [46,49], protocatechuic acid [50], catechin [51] and the aglycone of isorhamnetin-3-*O*-β-d-rutinoside [52].

#### 2.4.1. Survival Assay and ROS Accumulation

**Figure 7:**
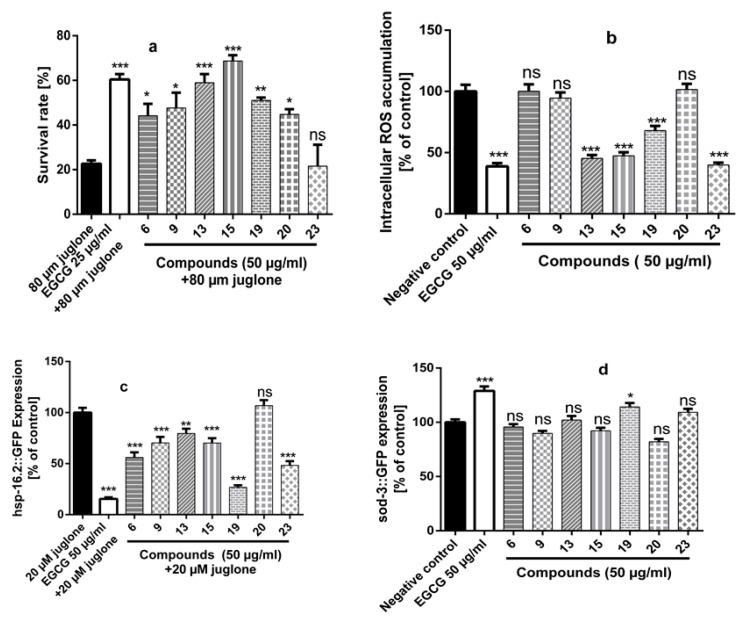
(**a**) Effect of compounds EGCG (epigallocatechin gallate) and compounds (**6**, **9**, **13**, **15**, **19**, **20**, and **23**) at concentration of (50 µg/mL) on the survival rate of nematodes N2 under oxidative stress caused by toxic dose of Juglone (80 µM). The pre-treatment of the worms with compounds (**6**, **9**, **13**, **15**, **19**, and **20**) (50 µg/mL) significantly improved the survival rates. (**b**) Intracellular reactive oxygen species (ROS) accumulation in N2 worms, using H_2_DCF-DA. Results are expressed as the percentage of fluorescence pixel intensity related to untreated control (100%). ROS accumulation was significantly reduced after pre-treatment of N2 worms with compounds (**13**, **15**, **19**, and **23**) (50 µg/mL). (**c**) hsp-16.2::GFP expression in the mutant TJ375 treated with EGCG, compounds (**6**, **9**, **13**, **15**, **19**, **20**, and **23**) after exposure to 20 µM Juglone. All treated worms, except compound (**20**), showed downregulation of hsp-16.2::GFP compared to the untreated control. (**d**) sod-3::GFP Expression in the mutant strains CF1553 after pre-treatment with EGCG, compounds (**6**, **9**, **13**, **15**, **19**, **20**, and **23**) at concentration of (50 µg/mL). Compound (**19**) showed significant up-regulation of sod-3 in mutants CF1553 related to the untreated control. The results are expressed as mean ± SEM from three independent experiments. Statistical significance of difference between untreated control and treated groups was analyzed by one-way analysis of variance followed by Bonferroni (post hoc) [ns (not significant) *p* > 0.05, * *p* < 0.05, ** *p* < 0.01, *** *p* < 0.0001].

The results (Figure 7a) described that the survival rates were significantly improved after pre-treating the worms with compounds (**6**, **9**, **13**, **15**, **19**, and **20**) to 44.17%, 47.67%, 58.97%, 68.67%, 51.05% and 44.74%, respectively, compared to 22.63% in the juglone treated control. Then we further evaluated the ability of these compounds to reduce intracellular ROS contents either by decreasing accumulation or scavenging them. The results (Figure 7b) showed compounds (**13**, **15**, **19**, and **23**) treated N2 worms had significant reduction in ROS level to 45.34%, 47.31%, 68.09%, and 39.90% after pre-treatment with compounds (**13**, **15**, **19**, and **23**) (50 μg/mL), respectively. The untreated control was set to 100%.

#### 2.4.2. Quantification of Stress Response Genes *hsp-16.2* and *sod-3*

The transgenic mutants TJ375 (hsp-16.2::GFP) were incubated with compounds (**6**, **9**, **13**, **15**, **19**, **20**, and **23**) for 72 h then a non-lethal dose of juglone (20 µM) was added. After 24 h, the expression of GFP was measured in the pharynx of the worms. Figure 7c shows that hsp-16.2::GFP expression levels was significantly reduced to 56.05%, 70.35%, 79.62%, 70.17%, 26.77%, and 48.31% (compared to the juglone treated control = 100%) after pre-treatment of the worms with compounds (**6**, **9**, **13**, **15**, **19**, and **23**) (50 µg/mL) respectively.

The expression of the *sod-3* gene was evaluated using the mutant CF1553. By measuring the intensity of the emitted fluorescence, it was noticed that among all tested compounds, pre-treatment with compound (**19**) resulted in higher levels of *sod-3* gene expression in comparison with untreated worms (Figure 7d).

It was clear that compound (**19**) among all tested compounds exerted high in vitro antioxidant activity, protected the worms against oxidative stress, reduced intracellular ROS, down-regulated *hsp 16.2* and up-regulated *sod-3*. On the other hand, compounds (**13** and **15**) which exerted weak in vitro antioxidant activity (DPPH IC_50_ > 100 µg/mL) significantly improved the survival of the worms under oxidative stress, diminished intracellular ROS contents and suppressed *hsp-16.2* expression under mild oxidative stress. These findings imply that, in addition to in vitro antioxidant ability, a tested compound can produce beneficial effects in vivo due to its interactions with cell-signaling processes, beside that the uptake of the compounds is prerequisite for producing systemic effects [53]. Further studies are thus needed to elucidate the exact mechanisms of these extracts and compounds.

## 3. Materials and Methods

### 3.1. Plant Materials

The fresh leaves and stem shoots of *Populus alba* L., fresh leaves and stem bark of *Salix subserrata* Willd. were collected in March 2016, from the vicinity of Banha city (Qalubeya governorate, Egypt) for *P. alba* and from the vicinity of Zagazig city (Sharkia governorate, Egypt) for *S. subserrata*. Voucher specimens for both plants were deposited in Department of Pharmacognosy, Faculty of Pharmacy, Zagazig University, Egypt.

### 3.2. Apparatus

The purity and the molecular weights of the isolated compounds were checked using GLC/MS (for compounds **1** and **5**) or ESI/MS. GLC/MS was carried out with HP 5890 Series II gas chromatograph (Hewlett Packard Inc., Böblingen, Germany) supplied with a ZB-5 column (length 30 m, internal diameter 0.25 mm and film thickness 0.25 μm) (Phenomenex, Aschaffenburg, Germany). The pressure of column’s head was set at 100 kPa. The carrier gas was helium and the injection temperature was 250 °C in split mode with ratio 1:200. Mass spectra were recorded with a Finnigan MAT SSQ-7000 quadrupole mass spectrometer (ThermoFinnigan, Bremen, Germany). The analysis of the data was carried out with Xcalibur 2.0.7 (Thermo-Finnigan, Bremen, Germany). For LC/MS ThermoFinnigan HPLC (Thermo Electron Corporation, Waltham, MA, USA) coupled with an LCQ-Duo ion trap mass spectrometer (ThermoQuest Corporation, Austin, TX, USA) with an ESI source (ThermoreQuest) was applied as described before [3]. ^1^H and^13^C NMR experiments were carried out using Varian AS500 MHz NMR spectrometer (Varian, CA, USA) at the operating frequencies of 500 and 125 MHz, respectively. UV absorbance was measured using a Shimadzu UV-1700 (Shimadzu, Kyoto 604-851, Japan).

### 3.3. Extraction and Fractionation

The air-dried shoots and leaf powders of *P. alba* and the stem bark and leaf powders of *S. subserrata* (2 kg, each) were separately extracted by cold maceration (4 × 6 L) using 80% methanol at room temperature. The corresponding filtrates were then combined, evaporated under reduced pressure to yield 112, 254, 350 and 450 g of viscous residues, respectively. The extracts were then separately suspended in 1 L of MeOH: H_2_O (1:9), successively partitioned using *n*-hexane, dichloromethane, ethyl acetate and n-butanol, dried over anhydrous sodium sulphate and concentrated under vacuum. As for *P. alba*, the shoot fractions had a yield of 15, 20, 20 and 22 g while the leaf fractions were 34, 26, 28 and 45 g. For *S. subserrata* stem bark fractions yielded 22, 15, 30 and 50 g and the leaf fractions were 85, 60, 50 and 65 g for *n*-hexane, dichloromethane, ethyl acetate and n-butanol fractions, respectively.

### 3.4. Chromatographic Isolation

Prior to chromatographic isolation, the fractions were investigated by TLC using the following solvent systems (A) *n*-hexane: dichloromethane (1.5:8.5), (B) *n*-hexane: ethyl acetate (1:1), (C) dichloromethane: methanol (9.5:0.5) (D) dichloromethane: methanol (9:1), and (E) ethyl acetate: methanol: water (30:5:4). Visualization of TLC plates was done by UV, anisaldehyde/H_2_SO_4_, ammonia, and ferric chloride solutions. The fractions that exhibited considerable antioxidant activity and major spots on TLC were further subjected to chromatographic isolation.

The dichloromethane (DF) (15 g) and ethyl acetate (EF) (17 g) soluble fractions of *P. alba* shoots were separately chromatographed on silica gel columns packed with n-hexane and the polarity was increased gradually with dichloromethane then methanol for DF and with ethyl acetate then methanol for EF. Similar fractions were pooled according to TLC chromatogram using solvent systems (A-C), concentrated and crystallized to give compounds (**1**–**4**) from DF and compounds (**5**–**11** and **16**) from EF.

The n-butanol soluble fraction *P. alba* leaf (35 g) was chromatographed on a silica gel column packed with ethyl acetate and the polarity was increased gradually using methanol. Fractions were investigated by TLC using solvent system (E), similar fractions were collected and crystallized to afford compounds (**12**–**15**).

The ethyl acetate soluble fraction (24 g) of *S. subserrata* bark was chromatographed on a silica gel column packed with dichloromethane and the polarity was increased gradually using methanol. Fractions were investigated by TLC using solvent systems (D and E) to afford compounds (**17**–**20**).

The n-butanol soluble fraction of *S. subserrata* leaf (35 g) was chromatographed on a silica gel column packed with ethyl acetate and the polarity was increased gradually with a mixture of ethyl acetate, methanol and distilled water with the ratio (30:5:1, 30:5:2, 30:5:3, 30:5:4, 30:7.5:4 and 30:10:4). Fractions were investigated by TLC using solvent system (E) to afford compounds (**21**–**24**).

### 3.5. In Vitro Antioxidant Assays

The Folin–Ciocalteu method was used to determine the total phenolic content and was carried out as previously detailed [54]. The DPPH and FRAP assays were conducted as described in [54]. For the DPPH assay, IC_50_ indicated the concentration (µg/mL) which exhibited 50% scavenging for DPPH radicals. The scavenging activity was measured according the following equation: DPPH scavenging effect (%) = [(A0 − A1)/A0] × 100 where A0 indicates the absorbance of the control reaction and A1 means the absorbance in the presence of the sample. The IC_50_ value was estimated by sigmoid non-linear regression [4].

### 3.6. In Vivo Antioxidant Activity

#### 3.6.1. *Caenorhabditis elegans*: Strains and Culture Conditions

The *C. elegans* strains used were N2 (wild type), CF1553 [muls84 [pAD76 (sod-3::GFP)], TJ375 (gpIs1[hsp-16-2::GFP]) and TJ356 [DAF-16::GFP]. The worms were sub-cultured on solid NGM (nematode growth media) plates seeded with living *Escherichia coli* (OP50) as a food for the worms and maintained at 20 °C in a temperature-controlled incubator. To obtain age synchronized worms, the lysis solution (0.5 mL of 5 M NaOH and 1 mL of 5% NaOCl) was added to the adults and the suspension was strongly shaken (5 min). The lysate was then centrifuged at 1200 rpm for 1 min and the supernatant was removed. To wash out the lysis solution, sterile water was added to the pellet and centrifuged at 1200 rpm for 1 min. The eggs were isolated apart from the debris using density gradient centrifugation. The obtained eggs (pellet) were re-suspended in M9 buffer for hatching and kept at 20 °C for 24 h. The obtained larvae (L1 stage) were moved to liquid S-medium seeded with living *E. coli* (O.D._600_ = 1.00) and used for the in vivo assays following the protocol of each experiment [4].

The employed *C. elegans* strains and *E. coli* OP50 in this work were obtained from the Caenorhabditis Genetics Center (CGC), University of Minnesota, Minneapolis, MN, USA.

#### 3.6.2. Survival Assay Under Oxidative Stress

Age synchronized N2 worms (L1 larval stage) grown in S-medium were separated into groups of 80 worms and incubated, except for the control group, with three different doses (50, 100 and 200 µg/mL) of *P. alba* leaf (PL), shoots (PS) and *S. subserrata* leaf (SL) extracts and one concentration (50 µg/mL) of the pure compounds (**6**, **9**, **13**, **15**, **19**, **20** and **23**) for 48 h. The oxidative stress was then induced by addition of the pro-oxidant juglone (80 µM) for another 24 h. The numbers of the dead and living worms were then recorded. The worms which did not react to gentle touching using a platinum wire were considered dead [4]. The experiment was done in triplicate and the results were represented as mean survival rate and compared with one-way analysis of variance (ANOVA) followed by Bonferroni (post-hoc).

#### 3.6.3. Intracellular ROS Accumulation

Age synchronized N2 worm (L1 larval stage, developed in S-medium) groups were treated, except the control group, with three different doses (50, 100 and 200 µg/mL) of PL, PS and SL extracts and one concentration (50 µg/mL) of the pure compounds (**6**, **9**, **13**, **15**, **19**, **20** and **23**) for 48 h. Afterwards, a solution of H2DCF-DA (50 µM, Fluka Chemie GmbH, Buchs, Switzerland) was added to the plates, which were then incubated for 1 h at 20 °C away from light [4]. The worms were then mounted on a glass slide and paralyzed by a drop of 10 mM sodium azide. Live images were taken for at least 30 worms per replicate using fluorescence microscopy at constant exposure time and with a 10× objective lens (BIOREVO BZ-9000, Keyence Deutschland GmbH, Neu-Isenburg, Germany; λEx 480/20 nm; **λ**Em 510/38 nm). The Image J software version 1.48 (National Institute of Health, Bethesda, MD, USA) was used for the determination of the worm’s body fluorescence. The assay was performed in three triplicates and the results were showed as mean fluorescence intensity (mean ± standard error of mean (SEM)) and compared by one-way ANOVA followed by Bonferroni (post-hoc) [4].

#### 3.6.4. Quantification of hsp-16.2:: GFP Expression

Age synchronized worms (TJ375,L1 stage, matured in S-medium) carrying a GFP reporter connected with hsp-16.2 were separated into groups and treated, except the control group, with PL, PS and SL extracts and the pure compounds (**6**, **9**, **13**, **15**, **19**, **20** and **23**) with the concentrations mentioned before for 72 h. Then 20 µM of the pro-oxidant juglone was added to the plates and the fluorescence of the worms were measured with fluorescence microcopy using a 20× objective lens after 24 h. The Image J software was used to determine densitometrically the relative fluorescence of the worm’s head. The results were presented as mean fluorescence intensity (mean ± SEM) and were compared by one-way analysis of variance followed by Bonferroni correction [4].

#### 3.6.5. Subcellular DAF-16: GFP Localization

Age-synchronized worms (TJ356, L1 stage, grown in S-medium) were treated, except the control group, with three different doses (50, 100 and 200 µg/mL) of PL, PS and SL extracts at 20 °C for 24 h in S-medium. Images were taken by a fluorescence microscope. According to the localization of the fusion DAF-16::GFP construct, worms were classified as showing cytosolic, intermediate, and nuclear Daf-16 localization [4].

#### 3.6.6. Quantification of sod-3:: GFP Expression

Age-synchronized worms (CF1553, L1 larval stage, matured in S media) carrying a GFP reporter combined with sod-3 gene were treated, except the control group, with PL, PS and SL extracts and the pure compounds (**6**, **9**, **13**, **15**, **19**, **20** and **23**) with the concentrations described before for 72 h, and then subjected to fluorescence microscopy, as described above. The Image J was used to measure densitometrically the posterior intestine relative fluorescence. The results were presented as mean fluorescence intensity (mean ± SEM) and compared by one-way analysis of variance (ANOVA) followed by Bonferroni correction [4].

## 4. Conclusions

To the best of our knowledge, the in vivo antioxidant activity for the *Populus alba* leaf, *Populus alba* shoots and *Salix subserrata* leaf extracts and the compounds aromadendrin, tremuloidin, salicin, isorhamnetin-3-*O*-β-D-rutinoside, gallocatechin, triandrin and chrysoeriol-7-*O*-glucuronide were determined for the first time. The obtained results can shed light on the further utilization of these extracts in medicinal field as promising natural antioxidant candidates and for further investigation of their in vivo activity and mechanisms as well.

## Figures and Tables

**Table 1:** In vitro antioxidant activities: TPC, TFC, DPPH and FRAP for the extracts of the leaf, shoots of *P. alba*, leaf and stem bark of *S. subserrata*, and their different fractions.

Plant Organ	Extract or Fraction	TPC	TFC	DPPH	FRAP
		mg GAE */g Extract	mg Quercetin/g Extract	(IC_50_ µg/mL)	(mM FeSO_4_/mg Extract)
*P. alba* leaf	Extract	139.55 ± 7.81	46.12 ± 1.19	27.45 ± 2.15 ^a^	12.12 ± 0.33 ^b^
	n-Hexane	43.53 ± 1.47	3.92 ± 0.49	>500	0.99 ± 0.01 ^b^
	Dichloromethane	72.48 ± 3.59	14.28 ± 1.12	63.15 ± 4.25 ^a^	8.40 ± 0.06 ^b^
	Ethyl acetate	329.10 ± 8.16	71.85 ± 4.56	18.90 ± 0.60 ^a^	14.20 ± 0.06 ^b^
	n-Butanol	133.79 ± 3.81	29.67 ± 2.15	41.10 ± 4.20 ^a^	9.99 ± 0.49 ^b^
*P. alba* stem	Extract	196.00 ± 4.41	41.16 ± 3.51	33.00 ± 1.00 ^a^	14.56 ± 0.54 ^b^
	n-Hexane	25.37 ± 2.74	3.18 ± 0.17	>500	1.52 ± 0.02 ^b^
	Dichloromethane	177.40 ±10.01	38.89 ± 1.73	42.00 ± 2.60 ^a^	11.04 ± 0.91 ^b^
	Ethyl acetate	244.70 ±11.31	61.01 ± 5.92	24.05 ± 1.95 ^a^	17.86 ± 0.61 ^b^
	n-Butanol	182.20 ± 2.58	41.90 ± 3.23	26.45 ± 0.05 ^a^	13.64 ± 0.28 ^b^
*S. subserrata* leaf	Extract	160.70 ± 9.04	34.60 ± 2.93	36.55 ± 2.15 ^a^	12.20 ± 0.01 ^b^
	n-Hexane	59.84 ± 1.64	9.87 ± 1.59	206.67 ± 1.31 ^a^	2.90 ± 0.12 ^b^
	Dichloromethane	110.02 ± 4.24	28.71 ± 1.9	132.69 ± 2.44 ^a^	5.91 ± 0.25 ^b^
	Ethyl acetate	253.13 ± 7.98	71.01± 5.92	20.15 ± 0.85 ^a^	19.89 ± 0.76 ^b^
	n-Butanol	160.03 ± 5.41	38.37± 2.57	37.10 ± 1.30 ^a^	13.08 ± 0.53 ^b^
*S. subserrata* stem bark	Extract	542.11 ± 4.88	119.16 ± 9.06	5.35 ± 0.05 ^a^	26.81 ± 2.55 ^b^
	n-Hexane	127.07 ± 5.11	14.79 ± 1.87	34.81 ± 0.54 ^a^	12.48 ± 0.92 ^b^
	Dichloromethane	257.73 ± 17.17	72.48 ± 3.35	23.12 ± 2.28 ^a^	15.69 ± 0.58 ^b^
	Ethyl acetate	530.13 ± 12.29	151.34 ± 8.31	4.55 ± 0.15 ^a^	26.30 ± 2.37 ^b^
	n-Butanol	429.66 ± 20.98	91.57 ± 6.35	9.30 ± 0.30 ^a^	26.89 ± 2.45 ^b^
Standard	EGCG ***	-	-	1.40 ± 0.05	36.18 ± 0.15

* Gallic acid equivalent. Values are mean ± standard deviation (SD) of three independent experiments. *** Epigallocatechin gallate. ^a^ *p* ˂ 0.05 significantly different from EGCG (DPPH). ^b^ *p* ˂ 0.05 significantly different from epigallocatechin gallate (EGCG) (FRAP).

**Table 2:** In vitro antioxidant activities: DPPH and FRAP for the isolated compounds.

Compound	DPPH	FRAP
	(IC_50_ µg/mL) *	(mM FeSO_4_/mg Extract)
**(1)**	nd	nd
**(2)**	>100	0.64 ± 0.01
**(3)**	>100	0.79 ± 0.02
**(4)**	>100	0.38 ± 0.01
**(5)**	>100	0.14 ± 0.01
**(6)**	18.64 ± 0.52	9.24 ± 0.99
**(7)**	8.06 ± 0.07	17.62 ± 1.12
**(8)**	3.59 ± 0.14	28.30 ± 1.94
**(9)**	>100	0.32 ± 0.01
**(10)**	>100	0.32 ± 0.01
**(11)**	>100	0.62 ± 0.02
**(12)**	>100	0.96 ± 0.02
**(13)**	>100	0.32 ± 0.01
**(14)**	>100	0.15 ± 0.01
**(15)**	>100	4.40 ± 0.30
**(16)**	2.27 ± 0.01	47.46 ± 4.62
**(17)**	3.92 ± 0.30	25.03 ± 2.5
**(18)**	3.25 ± 0.07	26.96 ± 0.25
**(19)**	2.99 ± 0.20	26.69 ± 1.92
**(20)**	29.50 ± 2.10	11.73 ± 0.91
**(21)**	5.04 ± 0.05	20.13 ± 0.13
**(22)**	>100	2.35 ± 0.16
**(23)**	>100	4.12 ± 0.12
**(24)**	>100	0.30 ± 0.03
EGCG **	1.40 ± 0.05	36.18 ± 0.15

* IC_50_ (μg/mL) was measured for the isolated pure compounds, which exhibited DPPH radical-scavenging activity more than 50% when tested in concentration of 100 μg/mL. Values are mean ± SD of three independent experiments. ** Epigallocatechin gallate. Nd = not determined.

**Table 3:** Linear correlation between total phenolic and flavonoid contents and antioxidant assays (DPPH and FRAP). Correlation significant at *p* < 0.05.

Experiment	Linear Correlation (R^2^)		
	TFC	FRAP	DPPH
TPC	96.3	99.61	88.43
DPPH	97.55	89.17	-
FRAP	96.26	-	-

## Supplementary Materials

The following are available online. UV, MS and NMR data of the isolated compounds from *Populus alba* L. and *Salix subserrata* Willd.

## Abbreviations

ABTS^+^, 2, 2′-Azino-bis-3-ethylbenzothiazoline-6-sulfonic acid; DPPH, 2,2-diphenyl-1-picryl-hydrazyl-hydrate; EDTA, ethylene diamine tetra acetic acid; FRAP, radical scavenging and ferric reducing antioxidant power; H_2_-DCFDA, 2′,7′-dichlorodihydrofluorescein diacetate; ORAC, Oxygen Radical Absorbance Capacity ROS, reactive oxygen species; SOD, superoxide dismutase; TLC, thin layer chromatography.
