# Supplementary material for: Phenolic Compounds from Populus alba L. and Salix subserrata Willd. (Salicaceae) Counteract Oxidative Stress in Caenorhabditis elegans
Source: Molecules. 2019 May 24;24(10):1999. doi: 10.3390/molecules24101999 (PMC6571762; doi:10.3390/molecules24101999)
Supplement: Supplementary file 1 [file molecules-24-01999-s001.pdf]

Phenolic compounds from *Populus alba* L. and *Salix subserrata* Willd. (Salicaceae) counteract oxidative stress in *Caenorhabditis elegans*

UV, MS and NMR data of the isolated compounds from *Populus alba* L. and *Salix subserrata* Willd.

Compounds (1-16) were isolated from *P. alba* and compounds (17-24) were isolated from *S. subserrata*.

Compound (1): White amorphous powder (2 g) with R_f value 0.4 (solvent system A). EI-MS, *m/z* (relative intensity %): 284(M^+, 2), 282 (3), 281 (4), 238 (4), 223 (2), 207 (20), 153 (6), 139 (8), 125 (14), 111 (24), 97 (47), 96 (15), 85 (16), 83 (37), 82 (26), 70 (20), 69 (41), 57 (64), 55 (57), 43 (100) and 41 (39). ^{1}H-NMR (500 MHz, $CDCl_3$): δ (ppm) 3.65 (2H, *t, J*= 6.5 Hz, H-1), 1.59 (2H, *m*, H-2), 1.27-1.37(33H, *m*, H - [3 - 18]) and 0.88 (3H, *t, J*= 7 Hz, H-19). ^{13}C-NMR (125 MHz, $CDCl_3$): δ (ppm) 63.11 (C-1), 32.82 (C-2), 31.92 (C-3), 29.35 - 29.65 (C - [4-16]), 25.73 (C-17), 22.69 (C-18) and 14.11(C-19). The spectral data of this compound are matching with the previously reported for **n-nonadecanol-1**.

Compound (2): Yellowish white amorphous powder (15 mg), with R_f 0.5, solvent system (B). UV: λ_{max} (MeOH) nm: 229 and 307. –ve ESI-MS, *m/z* (relative intensity %): 177 (100) $[M-H]^-$, MS^2 [177]: 163 (0.5) [coumaric acid-H]$^-$, 162 (6) $[M-H-15]^-$ and 145 (19) [coumaric acid-H-H_2O]$^-$.+ve ESI-MS, *m/z* (relative intensity %):179 (100%)

[M+H]$^+$, MS^2 [179]: 147 (100%) [coumaric acid + H - H_2O]$^-$. ^{1}H-NMR (500 MHz, CD_3OD): δ (ppm) 7.41 (2H, *d, J* = 8.5 Hz, H-2,6), 6.76 (2H,*d, J* = 8.5 Hz, H-3,5), 7.58 (1H,*d, J* = 15.5 Hz, H-7), 6.27 (1H, *d, J* = 15.5 Hz, H-8) and 3.75 (3H, s, -OCH_3). ^{13}C-NMR (125 MHz, CD_3OD): δ (ppm) 124.84 (C-1), 129.7 (C-2,6), 115.9 (C-3,5), 161.35 (C-4), 145.4(C-7), 112.7 (C-8), 168.48 (C-9) and 50.53(-OCH_3). By comparison of the data of this compound with the available literature, it is proved to be **trans-*p*-coumaric acid methyl ester.**

Compound (4): Colourless needles (500 mg) with R_f value 0.37 solvent (B), UV: λ_{max} (MeOH) nm: 284 and 330 (sh.). –ve ESI-MS, *m/z* (relative intensity %): 271 (100) [M-H]$^-$, MS^2 [271]: 227(2) [M-H-CO_2]$^-$, 177(21) [M – H - ring B]$^-$, 151 (100) [^{1,3}A]$^-$, and 107 (6) [^{1,3}A-CO_2]$^-$. +ve ESI-MS, *m/z* (relative intensity %): 273 (100) [M + H]$^+$, MS^2 [273]: 255 (2) [M + H - H_2O]$^+$, 231 (4) [M + H - 42]$^+$, 179 (6) [M + H - ring B]$^+$, 153 (54) [^{1,3}A]$^+$ and 147 (100) [^{0,4}B]$^+$. ^{1}H-NMR (500 MHz, CD_3OD): δ (ppm) 5.34 (1H, *dd, J* = 3,13Hz, H-2), 2.70 (1H, *dd, J* = 3,17 Hz, H-3 cis), 3.13 (1H, *dd, J* = 13,17 Hz, H-3 trans), 5.89 (1H, *d, J* = 2.5 Hz, H-6), 5.88 (1H, *d, J* = 2.5 Hz, H-8), 7.31 (2H, *d, J* = 8.5 Hz, H-2`,6`) and 6.82 (2H, *d, J* = 8.5 Hz, H-3`,5`). ^{13}C-NMR (125 MHz, CD_3OD):δ (ppm) 79.05 (C-2), 42.60 (C-3), 196.35 (C-4), 164.02 (C-5), 95.62 (C-6), 166.93 (C-7), 94.74 (C-8), 163.44 (C-9), 101.92 (C-10), 129.65 (C-1`), 127.60 (C-2`,6`), 157.58 (C-4`) and 114.89 (C-H-3`, 5`). Therefore, based on the spectroscopic data of compound (**4**) and by comparison with those reported previously, this compound was proved to be **naringenin.**

Compound (5): Shiny, white needle-shaped crystals (200 mg) with R_f value 0.49 solvent (C). EI-MS, *m/z* (relative abundance %):116 (M$^+$, 10), 115 (1), 98 (27), 97 (7),

83 (29), 80 (9), 71 (6), 70 (100), 69 (18), 67 (6), 57 (70), 55 (31), 54 (12), 53 (7), 45 (9), 44 (60) and 41 (63). ^{1}H-NMR (500 MHz, CD_3OD:$CDCl_3$, 9:1): δ (ppm) 3.67 (2H,*m*, H-1,2_{ax}), 1.72 (2H, *m*, H-3, 6_{eq}), 1.50 (2H, *m*, H-3, 6_{ax}), 1.59 (2H, *m*, H-4, 5_{eq}) and 1.28 (2H, *m*, H-4,5_{ax}). ^{13}C-NMR (125 MHz, CD_3OD:$CDCl_3$, 9:1): δ (ppm) 70.40 (C-1,2), 29.47 (C-3, 6) and 21.36 (C-4, 5), The data of compound (**5**) was matched with **trans-1,2-cyclohexanediol**.

Compound (6): Needle-shaped yellow crystals (50 mg) with R_f value 0.63 solvent (D). UV: λ_{max} (MeOH) nm: 290 and 340 (sh.). –ve ESI-MS, *m/z* (relative intensity %): 287 (100) $[M-H]^-$, MS^2 [287]: 269 (7) $[M – H - H_2O]^-$, 259 (100) $[M – H - CO]^-$ and 243 (14) $[M – H - CO_2]^-$. +ve ESI-MS, *m/z* (relative intensity %): 289 (100) $[M + H]^+$, MS^2 [289]: 271 (100) $[M + H - H_2O]^+$, 243 (71) $[M + H - H_2O - CO]^+$, 215 (0.4) $[M + H - H_2O -2CO]^+$, 195 (8) $[M + H - \text{ring B}]^+$, 163 (0.2) $[^{1,4}B]^+$, 153 (24) $[^{1,3}A]^+$ and 149 (2) $[^{0,2}A- H_2O]^+$. ^{1}H-NMR (500 MHz, CD_3OD): δ (ppm) 4.96 (1H, *d, J* = 11.5Hz, H-2), 4.52 (1H, *d, J* = 11.5 Hz, H-3), 5.85 (1H,*d, J* = 2Hz, H-6), 5.90 (1H, *d, J* = 2Hz, H-8), 7.34 (2H,*d, J* = 8.5 Hz, H-2`,6`) and 6.82 (2H,*d, J* = 8.5 Hz, H-3`,5`).^{13}C-NMR (125 MHz, CD_3OD): δ (ppm) 83.50 (C-2), 72.18 (C-3), 196.75 (C-4), 163.09 (C-5), 95.12 (C-6), 163.89 (C-7), 96.13 (C-8), 167.92 (C-9), 100.16 (C-10), 127.92 (C-1`), 128.91 (C-2`,6`), 157.76 (C-4`) and 114.69 (C-3`,5`). Compound (**6**) was confirmed to be **aromadendrin.**

Compound (7): Yellow amorphous powder (40 mg) with R_f value 0.56 solvent (D). UV: λ_{max} (MeOH) nm: 247 (band II) and 360 (band I), 382 (sh.). –ve ESI-MS, *m/z* (relative intensity %): 285 (100) $[M-H]^-$, MS^2 [285]: 285 (100), 257(2) [M-H-CO]+, 229 (1) $[M-H-2CO]^+$ and 151(1).+ve ESI-MS, *m/z* (relative intensity %): 287 (100) $[M+H]^+$, MS^2 [287]: 287 (100), 269 (2) $[M + H - H_2O]^+$, 241 (5) $[M + H - H_2O - CO]^+$, 231 (2) $[M + H - 2CO]^+$, 213 (2) $[M + H - H_2O - 2CO]^+$, 165 (2) $[^{0,2}A]^+$, 153 (1) $[^{1,3}A]^+$, 133 (1) $[^{1,3}B]^+$ and 121 (0.5) $[^{0,2}B]^+$. ^{1}H-NMR (500 MHz, CD_3OD): δ (ppm) 6.17 (1H, *d, J* = 2Hz, H-6), 6.37 (1H, *d, J* = 2Hz, H-8), 8.08 (2H, *d, J* = 9 Hz, H-2',6') and 6.90 (2H, *d, J* = 9 Hz, H-3',5'). ^{13}C-NMR (125 MHz, CD_3OD): δ (ppm) 146.58 (C-2), 135.67 (C-3), 175.92 (C-4), 161. 07 (C-5), 97.93 (C-6), 164.45 (C-7), 93.09 (C-8), 159.12 (C-9), 103.03 (C-10), 122.31 (C-1'), 129.23 (C-2',6'), 156.86 (C-4') and 114.87 (C-3',5'). Compound (7) was characterized as **kaempferol**.

Compound (8): Yellow amorphous powder (70 mg) with R_f value 0.48 solvent (D). UV: λ_{max} (MeOH) nm: 254 (band II) and 370 (band I). –ve ESI-MS, *m/z* (relative intensity %): 301 (100) $[M - H]^-$, MS^2 [301]: 301 (75) $[M - H]^-$, 273 (9) $[M – H - CO]^-$, 257 (12) $[M -H - CO_2]^-$, 229 (4) $[M – H - CO_2 CO]^-$, 211 (1), 193 (5) $[M – H - ring B]^-$, 179 (100) $[^{1,2}A]^-$, 169 (3), 151 (38) $[^{1,2}A - CO]^-$ or $[^{1,3}A]^-$, 121 (2) $[^{1,2}B]^-$ and 107 (2) $[^{1,2}A – CO - 2CO]^-$. +ve ESI-MS, *m/z* (relative intensity %): 303 (100) $[M + H]^+$, MS^2 [303]: 303 (100), 285 (11) $[M + H - H_2O]^+$, 257 (13) $[M + H - H2O - CO]^+$, 247 (4) $[M + H - 2CO]^+$, 229 (8) $[M + H - H2O - 2CO]^+$, 165 (4) $[^{0,2}A]^+$, 153(0.3) $[^{1,3}A]^+$,149(1) $[^{1,3}B]^+$, 137(3) $[^{0,2}B]^+$, and 121(1). ^{1}H-NMR (500 MHz, CD_3OD):δ (ppm)

6.17 (1H,*d, J* = 2Hz, H-6), 6.38 (1H,*d, J* = 2Hz, H-8), 7.72(1H,*d, J* = 2 Hz, H-2'), 6.88(1H,*d, J* = 8.5 Hz, H-5') and 7.621H,*dd, J* = 8.5,2 Hz, H-6').^{13}C-NMR (125 MHz, CD_3OD, δ ppm):147.33(C-2), 135.77(C-3), 175.88(C-4),161.00(C-5), 97.84(C-6), 164.25(C-7), 93.00(C-8),156.80(C-9),103.05(C-10), 122.70(C-1'), 114.79(C-2'),144.78 (C-3'),146.42 (C-4'),114.55 (C-5') and 120.23 (C-6').Compound (**8**) was identified as **quercetin**.

Compound (9): White amorphous powder (1.5 g) with R_f value 0.45 solvent (D).UV: λ_{max} (MeOH) nm:220 nm and 273nm.–ve ESI-MS, *m/z* (relative intensity %): 389(13) $[M-H]^-$and435(77)$[M+FA-H]^-$, MS^2[389]:121(100)[Benzoic acid-H]$^-$.+ve ESI-MS, m/z (relative intensity %):413 $[M + Na]^+$ (100).^{1}H-NMR (500 MHz, DMSO):δ (ppm)7.29 (1H,*dd, J* = 7.5,1.5Hz, H-3), 6.96(1H,*dt, J* = 7.5,1.5Hz,H-4), 7.15(1H,*dt, J* = 8.5,1.5Hz, H-5), 7.06 (1H,*dd, J* = 8.5,1.5Hz, H-6), 4.36 (1H,*dd, J* = 15, 5Hz, H-7_a), 4.10 (1H,*dd, J* = 15, 5Hz, H-7_b), 5.23 (1H, *d, J* = 8 Hz, H-1'), 5.05 (1H,*dd, J* = 9.5, 8 Hz, H-2'), 3.49-3.76 (5H, *m,* H[3'-6']), 7.97(2H,*dd, J* = 8,1.5 Hz, H-2'', 6''), 7.63(1H,*bt, J* = 7.5 Hz, H-4''), 7.50(2H,*bt, J* = 7.5 Hz, H-3'', 5''), 4.85(1H,*bt, J* = 5.5 Hz, 7-OH), 4.68(1H,*bt, J* = 5.5 Hz, 6'-OH), 5.47(1H, *d, J* = 5 Hz,3'-OH) and 5.31(1H, *d, J* = 5 Hz, 4'-OH).^{13}C-NMR (125 MHz, DMSO):δ (ppm) 153.81(C-1), 131.57 (C-2), 126.77 (C-3), 122.32 (C-4),127.76 (C-5), 114.40 (C-6), 57.70 (C-7), 98.71(C-1'), 74.64 (C-2'), 74.26 (C-3'), 70.33 (C-4'),77.66 (C-5'), 61.00 (C-6'),130.28 (C-1''), 129.72(C-2'',6''), 133.75(C-4''),128.50(C-3'',5''), 165.45(C-7''). Spectral data of UV, ESI-MS,^{1}H- and ^{13}C-NMR-APT of compound (**9**) were identical to **tremuloidin.**

Compound (10): White amorphous powder (2 g) with R_f value 0.41 solvent (D),EI-MS of the aglycone , *m/z*(relative abundance %): 414(100) [M^+ - sugar], 399(25), 396 (31), 381 (25), 329 (40), 303 (31), 273 (25), 255 (25), 21 (27), 159 (27), 145 (30), 119 (26), 107 (29), 105 (38), 95 (33), 81 (31), 69 (25), 57 (51) and 43 (88). ^{1}H-NMR (500 MHz, DMSO): δ (ppm) 3.46 (1H, *m*, H-3), 5.31 (1H, *br s.*, H-6), 0.63 (3H, *s*, H-18), 0.93 (3H, *s*, H-19), 0.88 (3H, *d, J* = 6.5 Hz, H-21), 0.78 (3H, *d, J* = 7 Hz, H-26), 0.79 (3H, *d, J* = 7 Hz, H-27), 0.81 (3H, *t*, *J* = 7 Hz, H-29), 4.20 (1H, *d*, *J*= 7.5 Hz, H-1 ` ), 2.87-3.10 (4H, *m*, sugar protons 2 ` , 3 ` , 4 ` , 5 ` ),3.63 (1H, m, H-6 ` *α*), 3.40 (1H, m, H-6 ` β) and 1 – 2.35 (18H, CH_2 of steroid nucleus and side chain). ^{13}C-NMR (125 MHz, DMSO): δ (ppm) 36.66 (C-1), 29.72 (C-2), 77.40 (C-3), 39.69 (C-4), 140.88 (C-5), 121.61 (C-6), 31.83 (C-7), 31.87 (C-8), 50.06 (C-9), 37.29 (C-10), 21.06 (C-11), 38.77 (C-12), 42.30 (C-13), 56.63 (C-14), 24.31 (C-15), 28.24 (C-16), 55.90 (C-17), 12.11 (C-18), 19.54 (C-19), 35.95 (C-20), 19.06 (C-21), 33.81 (C-22), 25.91 (C-23), 45.61 (C-24), 29.16 (C-25), 20.14 (C-26), 19.39 (C-27), 23.06 (C-28), 12.23 (C-29), 101.27 (C-1'), 73.90 (C-2'), 77.21 (C-3'), 70.52 (C-4'), 77.18 (C-5') and 61.53 (C-6'). Compound (**10**) was elucidated as ***β*-sitosterol -3-*O*-glucoside.**

Compound (11) Yellow amorphous powder (15 mg) with R_f value 0.39 solvent (D). UV: λ_{max} (MeOH) nm: 212, 226 and 308. –ve ESI-MS, *m/z* (relative intensity %): 423 (100) $[M-H]^-$, MS^2 [423]: 307 (0.5) $[M-H-116]^-$, 259 (1.6) $[M-H-164]^-$, 163 (73) for [coumaric acid-H]$^-$ =$[M-H-144]^-$ and 145(100) [coumaric acid-H-18]$^-$.+ve ESI-MS,

m/z (relative intensity %): 425 (65) $[M+H]^+$ and 447 (32) $[M+Na]^+$, MS^2 [425]: 309 (100) $[M+H-116]^+$, 263 (15) $[M+H-162]^+$ and 147 (72) $[M+H-116-162]^+$. ^{1}H-NMR (500 MHz, CD_3OD): δ (ppm) 3.87 (1H, *m,* H-1), 3.53 (1H, *m,* H-2), 1.28-1.79 (8H, *m,* H-[3-6]), 4.49 (1H, *d, J* = 8 Hz, H-1'), 5.05 (1H, *m,* H-2'), 3.41-3.83 (5H, *m,* H-[3'-6']), 7.46 (2H, *d, J* = 8.5 Hz, H-2'', 6''), 6.80 (2H, *d, J* = 8.5 Hz, H-3'', 5''), 7.65 (1H, *d, J* = 16 Hz, H-7'') and 6.39 (1H, *d, J* = 16 Hz, H-8''). ^{13}C-NMR (125 MHz, CD_3OD):δ (ppm) 78.16(C-1), 68.35 (C-2), 28.93 (C-3), 21.14 (C-4), 26.01 (C-5), 29.52 (C-6), 100.83 (C-1'), 77.39 (C-2'), 71.97 (C-3'), 69.58 (C-4'), 76.30 (C-5'), 60.88 (C-6'), 125.87 (C-1''), 129.67 C-2'', 6''), 159.62 (C-4''), 115.39 (C-3'', 5''), 145.11 (C-7''), 114.12 (C-8'') and 167.46 (C-9''). Spectral data of UV, ESI-MS, ^{1}H- and ^{13}C-NMR of compound (**11**) were consistent with that of **grandidentatin.**

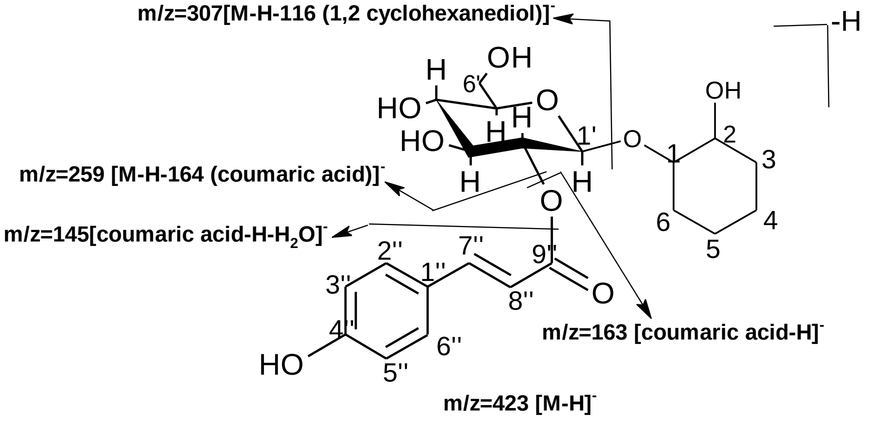

Figure 1. Proposed fragmentation of compound **(11).**

Compound (12): White amorphous powder (13 mg) with R_f value 0.35 solvent (D). UV: λ_{max} (MeOH) nm: 210 and 267. –ve. ESI-MS, *m/z* (relative intensity %): 327

(5) [M-H]⁻and 373 (50)[M+FA-H]⁻, MS2[327]: 285 (3)[M-H-acetyl residue(42)]⁻, 267(47)[M-H-acetic acid moiety (60)]⁻, 221 (18) [M-H-salicyl residue (106)]⁻ and 123 (100) [M-H-acetyl hexose residue (204)]⁻ [salicylalchol-H]⁻. +ve ESI-MS, *m/z* (relative intensity %): 351 (78) [M+Na]⁺. ^{1}H-NMR (500 MHz, CD_3OD):δ (ppm) 7.38 (1H, *dd, J* = 7.5, 1.5 Hz, H-3), 7.03 (1H, *dt, J* = 7.5,1 Hz, H-4), 7.22 (1H, *dt, J* = 8.5, 2Hz, H-5), 7.13 (1H, *dd, J* = 8, 1Hz, H-6), 4.58 (1H, *d, J* = 14 Hz, H-7_a), 4.52 (1H, *d, J* = 14 Hz, H-7_b), 5.06 (1H, *d, J* = 8 Hz, H-1'), 5.00 (1H, *dd, J* = 9.5, 8 Hz, H-2'), 3.47-3.92 (5H, *m,* H-[3'-6']) and 2.11 (3H, *s,* CH_3). ^{13}C-NMR (125 MHz, CD_3OD): δ (ppm) 154.39 (C-1), 130.41 (C-2), 127.50 (C-3), 122.27 (C-4), 128.02 (C-5), 114.56 (C-6), 58.55 (C-7), 99.17 (C-1'), 73.63 (C-2'), 74.52 (C-3'), 69.97 (C-4'), 76.87 (C-5'), 60.97 (C-6'), 170.57 (C=O), 19.60 (CH_3). Compound (**12**) was identified as **2-*O*-acetyl salicin.**

Compound (13): White amorphous powder (4 g) with R_f value 0.47 solvent (E). UV: λ_{max} (MeOH) nm: 210 and 268. –ve ESI-MS, *m/z* (relative intensity %): 285 (6) [M-H]⁻and 331 (21) [M+FA-H]⁻, MS^2 [285]: 123 (100) [M-H-162]⁻ , 121 (5) and 93 (2). +ve ESI-MS, *m/z* (relative intensity %): 309 (28) [M+Na]⁺. ^{1}H-NMR (500 MHz, DMSO): δ (ppm) 7.34 (1H, *dd, J* = 7.5, 2 Hz, H-3), 6.98 (1H, *dt, J* = 7.5, 1 Hz, H-4), 7.17 (1H, *dt, J* = 8,2 Hz, H-5), 7.07 (1H, *dd, J* = 8,1 Hz, H-6), 4.62 (1H, *dd, J* = 14,6 Hz, H-7_a), 4.44 (1H, *dd, J* = 14,6 Hz, H-7_b), 4.74 (1H, *d, J* = 8 Hz, H-1') and 3.21-3.70 (6H, *m,* H - [2'-6']). ^{13}C-APT-NMR (125 MHz, CD_3OD): δ (ppm) 155.12 (C-1), 131.95 (C-2), 127.63 (C3), 122.17 (C-4), 128.12 (C-5), 115.25 (C-6), 58.69 (C-7), 101.88 (C-

1'),73.85 (C-2'), 77.49 (C-3'), 70.10 (C-4'), 76.81 (C-5') and 61.11 (C-6'). Compound (**13**) was deduced as **salicin.**

Compound (14): White amorphous powder (16 mg) with R_f value 0.42 solvent (E).–ve ESI-MS, *m/z* (relative intensity %): 275(10) $[M-H]^-$ and 321 (40) $[M+FA-H]^-$ for $C_{12}H_{20}O_7$ and 277 (10) $[M-H]^-$, and 323 (15) $[M+FA-H]^-$ for $C_{12}H_{22}O_7$, MS^2 [275]: 257 (1) $[M-H-H_2O]^-$, 179 (100) $[glucose-H]^-$ = [M – H – 96 (1,2 cyclohexendiol residue)]⁻, 161 (38) [M – H – 114 (1,2 cyclohexendiol moiety)]⁻, 119 (32) [glucose – H - 60]⁻ and 89 (20) [glucose – H - 90]⁻, MS^2 [277]: 259 (0.4) $[M – H - H_2O]^-$, 241 (2) $[M – H - 2H_2O]^-$, 161 (100) [M – H – 116 (1,2 cyclohexanediol moiety)]⁻, 143 (4) $[M – H – 116 - H_2O]^-$, 125 (2) $[M – H – 116 - 2H_2O]^-$, 101(22)$[M-H-116-60]^-$. +ve ESI-MS, ***m/z*** (relative intensity %): 299 (100) $[M+Na]^+$ for $C_{12}H_{20}O_7$ and *m/z* 279(9) $[M+H]^+$ for $C_{12}H_{22}O_7$, MS^2 [279]: 279 (1) $[M+H]^+$, 261 (6) $[M + H - H_2O]^+$, 243 (100) $[M + H - 2H_2O]^+$, 225 (30) $[M + H - 3H_2O]^+$, 197 (7) $[M + H - 3H_2O - CO]^+$, 163 (96) [M + H – 116 (1,2 cyclohexanediol moiety)]⁺, 145 (53) $[M + H – 116 - H_2O]^+$, 127 (27) $[M + H – 116 -2H_2O]^+$ and 117 (53) [M + H – 162 (glucose)]⁺. ^1H-NMR (500 MHz, CD_3OD): δ (ppm) for compound (D4a): 3.81(1H, *m*, H-1), 3.82 (1H, *m*, H-2), 1.12-1.74 (8H, *m*, H - [3-6]), 4.36 (1H, *d, J* = 8 Hz, H-1') and 3.20 - 3.67 (6H, *m* H - [2'-6']); for compound (D4b): 4.27 (1H, *bs*, H-1), 3.92 (1H, *m*, H-2), 2.02 (2H, *m*, H-3), 5.86 (1H, *m*, H-4), 5.75 (1H, *m*, H-5), 1.7-1.9 (2H, *m*, H-6), 4.45 (1H, *d, J* = 7.5 Hz, H-1') and 3.20 - 3.67 (6H, *m*, H - [2'-6']).^{13}C-NMR (125 MHz, CD_3OD): δ (ppm) for compound (D4a): 77.94 (C-1), 69.49 (C-2), 26.26 (C-3), 21.01 (C-4), 21.64 (C-5), 30.00

(C-6), 100.77 (C-1'), 73.53 (C-2'), 76.44 (C-3'), 70.14 (C-4'), 76.46 (C-5') and 61.18 (C-6'); for compound (D4b): 74.66 (C-1), 67.65 (C-2), 22.58 (C-3), 130.78 (C-4), 124.31 (C-5), 25.74 (C-6), 101.68 (C-1'), 74.14 (C-2'), 76.51 (C-3'), 70.07 (C-4'), 76.56 (C-5') and 62.99 (C-6'). Compound (**14**) was elucidated as a mixture of 2-hydroxycyclohexyl-β-glucopyranoside (**a**) and 2-hydroxycyclohexenyl-β-glucopyranoside(**b**).

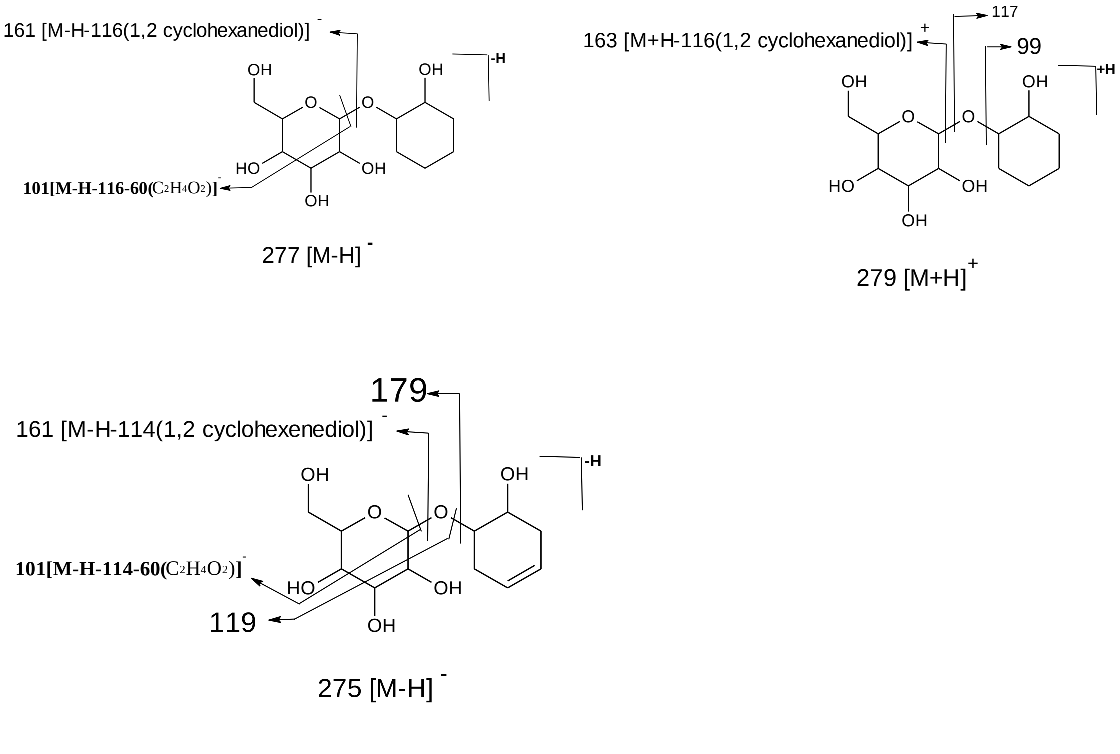

Figure 2. Proposed fragmentation of compound **14.**

Compound 15: Yellow amorphous powder (300 mg) with R_f value 0.40 solvent (E). UV: λ_{max} (MeOH) nm: 254, 354; (+NaOCH3): 261, 406; (+AlCl3): 268, 402; (+AlCl3+HCl): 268, 400; (+NaOAc): 273, 379; (+NaOAc + Boric acid): 255, 356. –ve

ESI-MS, *m/z* (relative intensity %): 623(100) $[M-H]^-$, MS^2 [623]: 595 (0.3) [M-H-CO], 477 (0.3) [M-H-146 (rhamnosyl)]$^-$, 316 (16), 315 (100) [M-H-308 (rutinosyl)]$^-$ = [aglycone - H]$^-$, 300 (17) [aglycone – H - CH_3]$^-$ and 271 (5) [aglycone – H - CO_2]$^-$. +ve ESI-MS, *m/z* (relative intensity %): 625 (79) $[M + H]^+$ and *m/z* 647 (100) $[M + Na]^+$. ^{1}H-NMR (500 MHz, CD_3OD): δ (ppm) 6.01 (1H, *d, J* = 2 Hz, H-6), 6.15 (1H, *d, J* = 2Hz, H-8), 3.92 (3H, *s*, OCH_3), 7.92 (1H, *d, J* = 2 Hz, H-2'), 6.84 (1H, *d, J* = 8.5 Hz, H-5'), 7.61 (1H,*dd, J* = 8.5,2 Hz, H-6'), 5.01(1H, *d, J* = 8 Hz, H-1''), 3.22-3.64 (6H, *m*, H-[2''-6'']), 4.51(1H, *d, J* = 1.5 Hz, H-1'''), 3.22-3.64 (4H, *m*, H-[2'''-5''']) and 1.12(3H, *d, J* = 6.5 Hz, H-6''').^{13}C-NMR (125 MHz, CD_3OD, δppm): 156.36 (C-2), 133.57 (C-3), 176.55 (C-4), 160.88 (C-5), 101.84 (C-6), 160.92 (C-7), 95.79 (C-8), 157.77 (C-9), 101.17 (C-10), 55.14 (OCH_3), 120.64 (C-1'), 112.90 (C-2'), 147.39 (C-3'), 151.33 (C-4'), 115.14 (C-5'), 122.60 (C-6'), 104.24 (C-1''), 74.46 (C-2''), 75.86 (C-3''), 70.78 (C-4''), 76.93(C-5''), 67.21 (C-6''), 101.17 (C-1'''), 70.65 (C-2'''), 72.49 (C-3'''), 70.11 (C-4'''), 68.37 (C-5''') and 16.48 (C-6'''). Compound 15 was confirmed to be **isorhamnetin-3-*O*-*β*-D-rutinoside.**

Compound (16): Light brown powder(16 mg) with R_f value 0.38 solvent (E).UV: λ_{max} (MeOH) nm: 230 and 321.–ve ESI-MS, *m/z* (relative intensity %): 153 (100) $[M-H]^-$, MS^2 [153]: 109 (100) [M-H-CO_2]$^-$.^{1}H-NMR (500 MHz, CD_3OD):δ (ppm) 7.30 (1H, *d, J* = 3 Hz, H-2), 6.65(1H, *d, J* = 8.5 Hz, H-5) and 6.79 (1H, *dd, J* = 3,8.5 Hz, H-6). ^{13}C-NMR (125 MHz, CD_3OD): δ (ppm) 118.03 (C-1), 120.41 (C-2), 148.47 (C-3),

154.19 (C-4), 115.53 (C-5), 116.16 (C-6) and 176.02 (C-7). Compound (**16**) was proved to be **protocatechuic acid (3,4 dihydroxybenzoic acid).**

Compound (17): White amorphous powder (1.5 g) with R_f value 0.85 solvent (E). UV: λ_{max} (MeOH) nm: 217 and 279.–ve ESI-MS, *m/z* (relative intensity %): 289 (100) [M-H], MS^2 [289]: 271 (4) [M – H - H_2O]$^-$, 247 (5) [M – H - CH_2CO (42)]$^-$, 245 (100) [M – H -CO_2]$^-$, 179 (13) [M – H – ring B (110)]$^-$, 163 (0.5) [^{1,4}B]$^-$, 137 (1) [^{1,3}A]$^-$ = [179 - CH_2CO]$^-$, 125 (2) [^{1,4}A]$^-$ and 109 (2) [ring B]$^-$ = [catechol-H]$^-$.+ve ESI-MS, *m/z* (relative intensity %): 291 (100) [M+H]$^+$, MS^2 [291]: 273 (22) [M + H - H_2O]$^+$, 249 (2) [M + H -CH_2CO (42 Da)]$^+$, 169 (4) [M + H – 122 Da]$^+$, 165 (92) [^{1,4}B]$^+$ = [M + H - phloroglucinol (126)]$^+$, 151 (27) [M + H - 140]$^+$, 147 (6) [M + H - 144]$^+$, 139 (97) [^{1,3}A]$^+$ = [M + H - 152]$^+$ and 123 (100) [^{0,2}B]$^+$. ^{1}H-NMR (500 MHz, CD_3OD): δ (ppm) 4.56 (1H, *d, J* = 7.5 Hz, H-2), 3.97 (1H, *m*, H-3), 2.84 (1H, *dd, J* = 5.5, 16 Hz, H-4α), 2.50 (1H, *dd, J* = 8,16 Hz, H-4β), 5.92 (1H, *d, J* = 2Hz, H-6), 5.85 (1H, *d, J* = 2Hz, H-8), 6.83 (1H, *d, J* = 2 Hz, H-2'), 6.76 (1H, *d, J* = 8 Hz, H-5') and 6.71 (1H, *dd, J* = 2,8 Hz, H-6').^{13}C-NMR (125 MHz, CD_3OD): δ (ppm) 81.42 (C-2), 67.39 (C-3), 27.09 (C-4), 156.14 (C-5), 94.87 (C-6), 156.39 (C-7), 94.09 (C-8), 155.48 (C-9), 99.40 (C-10), 130.79 (C-1'), 113.83 (C-2'), 144.79 (C-3'),144.81(C-4'), 114.66 (C-5') and 118.62 (C-6'). The spectral data of compound (**17**) were the same for **catechin**.

Compound (18): Yellow amorphous powder (7 mg) with R_f value 0.82 solvent (E). UV: λ_{max} (MeOH) nm: 212 and 279. –ve ESI-MS, *m/z* (relative intensity %): 577 (100) $[M-H]^-$, MS^2 [577]: 451 (39), 425 (100), 407 (34) and 289 (16). +ve ESI-MS, *m/z* (relative intensity %): 579 (100) $[M + H]^+$, MS^2 [579]: 453 (95), 435 (41), 427 (49), 409 (100) and 291 (21). Compound (**18**) was identified by ESI-MS as (**epi)catechin-(epi)catechin.**

Compound (19): Yellowish white amorphous (1 g) with R_f value 0.80 solvent (E). UV: λ_{max} (MeOH) nm: 214 and 270. –ve ESI-MS, *m/z* (relative intensity %): 305 (50) $[M - H]^-$, MS^2 [305]: 287 (11) $[M – H - H_2O]^-$, 261 (42) $[M – H - CO_2]^-$, 179 (100) $[M – H - \text{ring B } (126)]^-$, 137 (15) $[^{1,3}A]^-$ = $[179 - CH_2CO]^-$ and 125 (22) [ring B (pyrogallol)]$^-$= $[^{1,4}A]^-$.+ve ESI-MS, *m/z* (relative intensity %):307(42) $[M+H]^+$. MS^2[307]: 289 (39) $[M+H-H_2O]^+$, 263(0.2) $[M+H-CO_2]^+$, 181(13)$[^{1,4}B]^+$= [M+H-phloroglucinol (126)]$^+$, 169(9) $[M+H-138]^+$, 163(5) $[M+H-144]^+$,151(78) $[M+H-156]^+$ and 139 (100) $[^{1,3}A]^+$ $[M+H-168]^+$ or $[^{0,2}B]^+$. ^{1}H-NMR (500 MHz, CD_3OD): δ (ppm) 4.52 (1H, *d, J* = 7 Hz, H-2), 3.96 (1H, *m*, H-3), 2.82 (1H, *dd, J* = 5.5,16.5 Hz, H-4α), 2.49 (1H, *dd, J* = 8,16.5 Hz, H-4β), 5.91 (1H, *d, J* = 2.5 Hz, H-6), 5.85 (1H, *d, J* = 2.5 Hz, H-8) and 6.39 (2H, *s*, H-2',6'). ^{13}C-NMR (125 MHz, CD_3OD): δ (ppm) 81.44 (C-2), 67.33 (C-3), 26.67 (C-4), 156.16 (C-5), 94.83 (C-6), 156.39 (C-7), 94.08 (C-8), 155.40 (C-9), 99.29 (C-10), 130.13 (C-1'), 105.75 (C-2',6'), 145.42 (C-3',5') and 132.46 (C-4'). The spectral data of compound (**19**) were matched with that of **gallocatechin.**

Compound (20): White needles (1.2 g) with R_f value 0.53 solvent (E). UV: λ_{max} (MeOH) nm: 206 and 260. The –ve ESI-MS spectrum showed the following fragments at *m/z* (relative abundance %): 311 (10) $[M-H]^-$ and 357 (100) $[M + FA - H]^-$, MS^2 [357]: 311 (2) $[M-H]^-$, 225 (11) $[M – H - 86]^-$, 179 (100) $[glucose - H]^-$ = [M – H -132 (dehydrated p-coumaryl)]$^-$, 161 (2) [M – H – 150 (p-coumaryl)]$^-$ and 143 (1) $[M – H - 168]^-$. The +ve ESI-MS spectrum showed the following fragments at *m/z* (relative abundance %): 335 (17) $[M+Na]^+$. MS^2 [335]: 275 (13) $[M+Na-60]^+$, 203 (100) [M+Na-132 (dehydrated *p*-coumaryl)]$^+$,155 (21) [M+Na -180 (glucose)]$^+$ and 133 (9) $[M+Na-202]^+$. ^{1}H-NMR (500 MHz, CD_3OD): δ (ppm) 7.24 (2H, *d, J* = 8.5 Hz, H-2,6), 6.72 (2H, *d, J* = 8.5 Hz, H-3,5), 6.57 (1H, *brd, J* = 16 Hz, H-7), 6.16 (1H, *ddd, J* = 16,6.5,6.5 Hz, H-8), 4.47 (1H, *ddd, J* = 12,6.5,1.5 Hz, H-9a), 4.27 (1H, *ddd, J* = 12,6.5,1.5 Hz, H-9b), 4.35 (1H, *d, J* = 8 Hz, H-1'), 3.19-3.37 (4H, *m*, H-[2'-5']), 3.85-3.89 (1H, *m*, 6'a) and 3.65-3.69 (1H, *m*, 6'b). ^{13}C-NMR (125 MHz, CD_3OD): δ (ppm) 128.32 (C-1), 127.41 (C-2,6), 114.92 (C-3, 5), 157.04 (C-4), 132.72 (C-7), 121.91 (C-8), 69.66 (C-9), 101.70 (C-1'), 73.70 (C-2'), 76.67 (C-3'), 70.27 (C-4') and 61.37 (C-6'). Compound (**20**) was characterized as **4-hydroxy-cinnamyl-*O*-β-D-glucopyranoside (triandrin).**

Compound (21): Yellow amorphous powder (9 mg) with R_f value 0.50 solvent (E).UV: λ_{max} (MeOH) nm:258, 363; (+$NaOCH_3$): 277, 437; (+$AlCl_3$): 269, 425; (+$AlCl_3$+HCl): 271, 404; (+NaOAc): 269,400; (+NaOAc + Boric acid): 269, 422. –ve ESI-MS, *m/z* (relative intensity %): 479 (100) $[M-H]^-$, MS^2 [479]: 461 (3) $[M-H-18]^-$, 433 (0.2)

[M-H-18-28]$^-$, 317 (27) [M-H-162]$^-$, 316 (100), 289 (0.3) [aglycone - 28]$^-$, 245 (0.2) [aglycone-28-44]$^-$, 179 (3) [^{1,2}A]$^-$and 151 (1) [^{1,2}A-CO]$^-$. +ve ESI-MS, *m/z* (relative intensity %): 481 (55) [M+H]$^+$ and 503 (30) [M+Na]$^+$, MS^2 [481]: 319 (100) [M+H-162]$^+$. ^{1}H-NMR (500 MHz, DMSO): δ (ppm) 6.03 (1H, *d, J* = 3 Hz, H-6), 6.21 (1H, *d, J* = 3Hz, H-8),7.17 (2H, *d, J* = 4Hz ,H-2',6'), 5.25 (1H, *d, J* = 7.5Hz, H-1'') and 3.08-3.63 (6H, *m*, H-[2''-6'']). Compound (**21**) was elucidated as **myricetin-3-*O*-*β*-D-glucoside.**

Compound (22): White crystals (600 mg) with R_f value 0.32 solvent (E). UV: λ_{max} (MeOH) nm: 227, 272 and 347. –ve ESI-MS, *m/z* (relative intensity %): 203 (36) [M-H]$^-$. and 407 (100) [2M-H]$^-$, MS^2 [407]: 203 (100) [M-H]$^-$ and 159 (0.5) [M-H-44]$^-$.+ve ESI-MS, *m/z* (relative intensity %): 205 (100) [M+H]$^+$, MS^2 [205]: 188 (100) [M+H-17]$^+$. ^{1}H-NMR (500 MHz, DMSO): δ (ppm) 10.91 (1H, *s*, H-1), 7.19 (1H, *d, J* = 2.5 Hz, H-2), 7.54 (1H, *dd, J* = 1,7.5 Hz, H-5), 6.95 (1H, *dt, J* = 1,8 Hz, H-6), 7.04 (1H, *dt, J* = 1,8.5 Hz, H-7), 7.32 (1H, *dd, J* = 1,8.5 Hz, H-8), 2.94 (1H, *dd, J* = 9,15 Hz, H-10a), 3.92 (1H, *dd, J* = 4,15.5 Hz, H-10b) and 3.43 (1H, *dd, J* = 4,9 Hz, H-11). ^{13}C-NMR (125 MHz, DMSO): δ (ppm) 124.46 (C-2), 110.10 (C-3), 127.71 (C-4), 118.81 (C-5), 118.69 (C-6), 121.31 (C-7), 111.75 (C-8), 136.77 (C-9), 27.60 (C-10), 55.22 (C-11) and 170.46 (C-12). Compound (**22**) was deduced as **tryptophan.**

Compound (23): Yellow amorphous powder (750 mg) with R_f value 0.29 solvent (E).UV: λ_{max} (MeOH) nm 266, 345; (+$NaOCH_3$): 264, 388; (+$AlCl_3$): 272, 377; (+$AlCl_3$+HCl): 269, 350; (+NaOAc): 266, 345; (+NaOAc + Boric acid): 266, 345. –ve ESI-MS, *m/z* (relative intensity %): 475(85) [M-H]$^-$, MS^2 [475]: 299 (100) [M-H-176]$^-$

and 285 (1) [aglycone-14]$^{-}$.+ve ESI-MS, *m/z* (relative intensity %): 477 (100) $[M+H]^{+}$and *m/z* 499 (10) $[M+Na]^{+}$, MS^2[477]: 301(100) $[M+H-176]^{+}$. ^{1}H-NMR (500 MHz, DMSO): δ (ppm) 6.94 (1H, *s*, H-3), 12.95 (1H, *s*,5-OH), 6.41 (1H,*bs*, H-6), 6.82 (1H, *bs*, H-8), 3.86(3H, *s*, OCH_3), 7.54 (1H, *bs*, H-2'), 6.93 (1H, *d, J* = 8 Hz, H-5'), 7.55 (1H, *bd, J* = 8Hz, H-6'), 5.08 (1H, *d, J* = 7.5 Hz, H-1''), 3.21-3.28 (3H, *m*, H-[2''-4'']), 3.64 (1H, *d, J* = 10 Hz, H-5'').^{13}C-NMR (125 MHz, DMSO): δ (ppm) 164.57 (C-2), 103.80 (C-3), 182.43 (C-4), 161.50 (C-5), 99.96 (C-6), 163.43 (C-7), 95.27 (C-8), 157.33 (C-9), 105.70 (C-10), 56.43 (OCH_3), 124.70 (C-1'), 110.72 (C-2'), 148.53 (C-3'), 153.71 (C-4'), 116.24 (C-5'), 120.93 (C-6'), 99.96 (C-1''), 73.39 (C-2''), 76.84 (C-3''), 72.37 (C-4''), 74.54 (C-5'') and 172.93 (C-6''). Compound (**23**) was elucidated as **chrysoeriol-7-*O*-glucuronide.**

Compound (24): White amorphous powder (6 mg) with R_f value 0.19 solvent (E). UV: λ_{max} (MeOH) nm: 205, 258.–ve ESI-MS, *m/z* (relative intensity %):164(100) $[M-H]^{-}$, MS^2 [164]: 147 (100) $[M-H-NH_3]^{-}$, 119 (0.2) $[M-H-NH_3-CO]^{-}$.+ve ESI-MS, *m/z* (relative intensity %): 166 (100) $[M+H]^{+}$, MS^2 [166]: 120 (100) $[M+H-H_2O-CO]^{+}$. Compound (**23**) was identified as **phenyl alanine.**
